# Neuronal Calcium Sensor 1 is up‐regulated in response to stress to promote cell survival and motility in cancer cells

**DOI:** 10.1002/1878-0261.12678

**Published:** 2020-04-28

**Authors:** Henrike K. Grosshans, Tom T. Fischer, Julia A. Steinle, Allison L. Brill, Barbara E. Ehrlich

**Affiliations:** ^1^ Department of Pharmacology Yale University School of Medicine New Haven CT USA; ^2^ Institute of Pharmacology Heidelberg University Germany; ^3^ Department of Cellular and Molecular Physiology Yale University School of Medicine New Haven CT USA; ^4^ Present address: University of Freiburg School of Medicine Germany; ^5^ Present address: University of Muenster School of Medicine Germany

**Keywords:** Ca^2+^ binding protein, Ca^2+^ signaling, NFκB, PKB/Akt, tumor progression

## Abstract

Changes in intracellular calcium (Ca^2+^) signaling can modulate cellular machinery required for cancer progression. Neuronal calcium sensor 1 (NCS1) is a ubiquitously expressed Ca^2+^‐binding protein that promotes tumor aggressiveness by enhancing cell survival and metastasis. However, the underlying mechanism by which NCS1 contributes to increased tumor aggressiveness has yet to be identified. In this study, we aimed to determine (a) whether NCS1 expression changes in response to external stimuli, (b) the importance of NCS1 for cell survival and migration, and (c) the cellular mechanism(s) through which NSC1 modulates these outcomes. We found that NCS1 abundance increases under conditions of stress, most prominently after stimulation with the pro‐inflammatory cytokine tumor necrosis factor α, in a manner dependent on nuclear factor kappa‐light‐chain‐enhancer of activated B cells (NFκB). We found that NFκB signaling is activated in human breast cancer tissue, which was accompanied by an increase in *NCS1* mRNA expression. Further exploration into the relevance of NCS1 in breast cancer progression showed that knockout of NCS1 (NCS1 KO) caused decreased cell survival and motility, increased baseline intracellular Ca^2+^ levels, and decreased inositol 1,4,5‐trisphosphate‐mediated Ca^2+^ responses. Protein kinase B (Akt) activity was decreased in NCS1 KO cells, which could be rescued by buffering intracellular Ca^2+^. Conversely, Akt activity was increased in cells overexpressing NCS1 (NCS1 OE). We therefore conclude that NCS1 acts as cellular stress response protein up‐regulated by stress‐induced NFκB signaling and that NCS1 influences cell survival and motility through effects on Ca^2+^ signaling and Akt pathway activation.

## Graphical abstract

This study investigates the underlying mechanism by which the calcium (Ca^2+^)‐binding protein NCS1 promotes increased tumor aggressiveness. We characterize NCS1 as a stress response protein that is up‐regulated by exogenous stressors, including the cytokine tumor necrosis factor α, through a nuclear factor kappa‐light‐chain‐enhancer of activated B cells‐dependent mechanism. Increased NCS1 expression leads to disrupted intracellular Ca^2+^ signaling and activation of the protein kinase B pathway, leading to increased cell survival and migration.

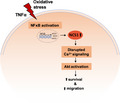

**Abbreviations**
Akt: protein kinase B
Ca^2+^: calcium
ER: endoplasmic reticulum
InsP3: inositol 1,4,5‐trisphosphate
InsP3R: inositol 1,4,5‐trisphosphate receptor
IκBα: nuclear factor of kappa‐light polypeptide gene enhancer in B‐cell inhibition, alpha
NCS1: neuronal calcium sensor 1
NFκB: nuclear factor kappa‐light‐chain‐enhancer of activated B cells
p‐Akt: phosphorylated Akt
PI3K: phosphoinositide‐3‐kinase
PP2Ac: catalytic subunit of protein phosphatase 2A
tBHP: tert‐butylhydroperoxide
TG: thapsigargin
TNF‐α: tumor necrosis factor alpha

## 1 Introduction

Disrupted intracellular calcium (Ca^2+^) signaling leads to a variety of pathological conditions (Carafoli, 2002; Clapham, 2007), including tumor development and progression (Monteith *et al.*, 2017). Under resting conditions, free cytoplasmic Ca^2+^ levels are kept at low concentrations (100 nm), such that following defined stimuli, Ca^2+^ levels can be raised and used as an effective second messenger. Ca^2+^ transients cause a broad variety of outcomes not only due to the tightly regulated spatial and temporal occurrence of Ca^2+^ signals, but also due to the existence of Ca^2+^‐binding proteins. Upon binding of Ca^2+^, these proteins can change conformation and interact with other effector proteins. As such, Ca^2+^‐binding proteins are essential for regulation of cellular homeostasis.

Changes in the expression levels or functionality of Ca^2+^‐binding proteins can lead to numerous diseases, including cancer. A well‐established example of this is the S100 family of Ca^2+^‐binding proteins, which promote tumor aggressiveness in several different cancer types, such as breast, lung, prostate, colorectal, and liver cancers (Bresnick *et al.*, 2015). Interestingly, S100A7 is not expressed in healthy breast tissue, but is highly expressed in multiple types of breast cancer and activates several pro‐survival pathways, including those that rely upon signaling of nuclear factor kappa‐light‐chain‐enhancer of activated B cells (NFκB) and protein kinase B (Akt) (Bresnick *et al.*, 2015; Salama *et al.*, 2008). The expression of another member of the S100 family, S100A6, is induced by tumor necrosis factor α (TNF‐α), a strong activator of NFκB (Joo *et al.*, 2003), and promotes cell proliferation and migration through activation of the Akt pathway (Li *et al.*, 2014). These findings provide insights into how Ca^2+^‐binding proteins can affect tumor progression. Here, we focus on the Ca^2+^‐binding protein neuronal calcium sensor 1 (NCS1), which shares multiple characteristics with the S100 protein family. Like S100 proteins, NCS1 belongs to the EF‐hand superfamily of Ca^2+^‐binding proteins (Burgoyne, 2007; Donato *et al.*, 2013), is increased in cancerous tissues, and contributes to a more aggressive phenotype of these tumors (Apasu *et al.*, 2019; Moore *et al.*, 2017; Moore *et al.*, 2018; Schuette *et al.*, 2018). It binds Ca^2+^ with high affinity and contains four EF‐hand domains, three of which bind Ca^2+^, and one of which, in the N‐terminal domain, is unable to bind Ca^2+^ (Boeckel and Ehrlich, 2018; Bourne *et al.*, 2001).

NCS1 was first thought to be expressed only in neuronal cells, but is now known to be expressed and to have essential functions in almost every tissue type (Burgoyne, 2007). Besides its ability to bind and buffer intracellular Ca^2+^, NCS1 can modulate numerous effector proteins, including the inositol 1,4,5‐trisphosphate receptor (InsP3R) (Schlecker *et al.*, 2006), phosphatidylinositol‐4‐kinase IIIβ (Graham and Burd, 2011), and calcineurin (Schaad *et al.*, 1996). These interactions, the capacity to bind and buffer Ca^2+^_,_ and its broad tissue distribution help to explain its diverse set of functions. For example, in neurons NCS1 is involved in Ca^2+^ signal transduction (Dragicevic *et al.*, 2014; Weiss *et al.*, 2010), exocytosis (Koizumi *et al.*, 2002), membrane trafficking (Zhao *et al.*, 2001), cell survival (Nakamura *et al.*, 2006), and hippocampal learning (Sippy *et al.*, 2003). In the heart, NCS1 plays an essential role in development and function, as it regulates cardiomyocyte contraction and contributes to the stress tolerance of cardiac cells (Nakamura *et al.*, 2011; Nakamura *et al.*, 2016; Nakamura‐Nishitani and Wakabayashi, 2014). NCS1 is also involved in the development and progression of breast and liver cancer (Apasu *et al.*, 2019; Moore *et al.*, 2017; Moore *et al.*, 2018; Schuette *et al.*, 2018). Whereas NCS1 levels do not significantly differ among healthy individuals, they rise during the development of aggressive tumors, and increased levels are correlated with worse patient outcome (Moore *et al.*, 2017; Moore *et al.*, 2018; Schuette *et al.*, 2018). High NCS1 levels promote tumor aggressiveness by enhancing cell survival and migration in 2D and 3D cell culture models and in mice (Apasu *et al.*, 2019). However, the underlying cellular mechanisms for these observations are not yet determined.

In this study, we aimed to investigate the mechanisms by which NCS1 enhances tumor progression and aggressiveness. Our primary goal was to determine the specific extracellular conditions and intracellular signaling pathway(s) that lead to increased NCS1 expression in cancer cells. For this purpose, we explored whether NCS1 expression levels change in response to external stimuli *in vitro* and investigated the translational relevance of the identified signaling mechanism in human cancer. We determined the importance of NCS1 for cell survival and migration using a model of breast cancer cells (MDA‐MB231) lacking NCS1 expression. Finally, we investigated which cellular mechanisms are used by NSC1 to affect cell survival and migration, focusing on Ca^2+^ homeostasis, InsP3‐mediated Ca^2+^ signaling, and phosphatidylinositol 3‐kinase (PI3K)‐protein kinase B/Akt pathway (Akt pathway) activation. Overall, we describe a novel mechanism through which NCS1 functions as a stress response protein to promote cell survival and motility.

## 2 Methods

### 2.1 Cell culture

MDA‐MB231 human breast cancer and SHSY5Y human neuroblastoma cell lines were obtained from the American Type Culture Collection (ATCC, Manassas, VA, USA). ATCC validates all cell lines by short‐tandem repeat analysis. MDA‐MB231 cell lines were maintained at 37 °C with 5% CO_2_ in Dulbecco's modified essential medium (DMEM) supplemented with 10% FBS, 1% l‐glutamine, and 1% penicillin/streptomycin. MDA‐MB231 cells stably overexpressing NCS1 (Moore *et al.*, 2017) were cultured in the medium described above plus 1 mg·mL^−1^ G418. SHSY5Y cells were maintained at 37 °C with 5% CO_2_ in DMEM/F12 medium supplemented with 10% FBS, 1% nonessential amino acids, and 1% penicillin/streptomycin.

### 2.2 *In vitro* treatments

To study the effect of cellular stressors on NCS1 expression, SHSY5Y cells were treated with various stimuli. To specifically activate the transcription factor NFκB, cells were treated with 10 ng·mL^−1^ TNF‐α (Sigma‐Aldrich, St. Louis, MO, USA) for 24–36 h. For NFκB inhibition, 1 μm Bay 11‐7082 (Sigma‐Aldrich) was used for 24 h. Bay 11‐7082 was either applied alone or together with TNF‐α. To induce oxidative stress, cells were treated with 10 μm tert‐butylhydroperoxide (tBHP) for 20 h. To buffer intracellular Ca^2+^, MDA‐MB231 cells were treated with 1 μm BAPTA‐AM for 30 min. To induce high extracellular Ca^2+^ levels, we added 5 mm Ca^2+^ to the cell culture medium for 24 h. To induce endoplasmic reticulum (ER) stress, cells were treated with 1 μm thapsigargin (TG) for 24 h.

### 2.3 Quantitative real‐time PCR

RNA was extracted using an RNeasy Mini Kit (QIAGEN, Hilden, Germany) according to the manufacturer's protocol. Briefly, cells were lysed and homogenized using QIAshredder spin columns (QIAGEN) and RNA was bound to a RNeasy Mini Spin Column. To eliminate genomic DNA contamination, on‐column DNA digestion was performed using RNAase‐Free DNAse I in buffer RD (QIAGEN). After several washing steps, RNA was eluted. RNA concentration and quality were assessed with spectrophotometry (NanoDrop; Thermo Scientific, Waltham, MA, USA). Using a High‐Capacity cDNA Reverse Transcription Kit (4368814; Thermo Fisher Scientific), 1 μg RNA was then reverse‐transcribed to cDNA in a total reaction volume of 20 μL. Subsequently, the cDNA reaction was diluted with a dilution factor of 1 : 3. Real‐time quantitative PCR was performed on MicroAmp Fast 96‐well reaction plates (Applied Biosystems, Waltham, MA, USA) using 1 μL of the diluted cDNA reaction per well and Power SYBR Green PCR Master Mix (Applied Biosystems) in a 7300 Real‐Time PCR System (Thermo Fisher Scientific). Data were analyzed using the ΔΔ*C*_t_ method (Livak and Schmittgen, 2001) and ribosomal protein 18S used as a housekeeping gene. The following primers were used at a concentration of 5 µm: NCS1 (forward, 5′‐GATGCTGGACATTGTGGATG‐3′; reverse, 5′‐CTTGGAACCCTCCTGGAACT‐3′), and 18S (forward, 5′‐TTCGAACGTCTGCCCTATCAA‐3′; reverse, 5′‐ATGGTAGGCACGGCGACTA‐3′).

### 2.4 Western blot analysis

#### 2.4.1 Whole‐cell lysates

Cells were lysed in ice‐cold mammalian protein extraction reagent (PER) buffer (Thermo Fisher Scientific) supplemented with a protease inhibitor, sodium fluoride, and sodium orthovanadate (Santa Cruz Biotechnology, Dallas, TX, USA).

#### 2.4.2 Extraction of nuclear and cytosolic fractions

To obtain cytosolic and nuclear fractions, NE‐PER nuclear and cytoplasmic extraction reagents (Thermo Fisher Scientific) were used according to the manufacturer's protocol. Cells were harvested with TrypLE, centrifuged, and washed in PBS. A reagent ratio of 200 cytoplasmic extraction reagent I (CER I): 11 cytoplasmic extraction reagent II (CER II): 100 nuclear extraction reagent (NER) was used. Ice‐cold CER I containing protease inhibitor was added to the cell pellet and vortexed to fully suspend the cell pellet. The cell suspension was incubated for 10 min on ice. Ice‐cold CER II was added, vortexed, and incubated for 1 min on ice. After an additional vortexing step and following centrifugation, the cytoplasmic extract was obtained from the supernatant. Ice‐cold NER was added to the insoluble fraction (pellet) containing the nuclei. After several vortexing steps, the suspension was centrifuged and the nuclear protein fraction was obtained from the supernatant.

All protein concentrations were determined using the Pierce BCA Protein Assay Kit (Thermo Fisher Scientific). SDS/PAGE was performed with 10–30 μg protein. Protein was transferred to a polyvinylidene difluoride membrane (GE Healthcare, Arlington Heights, IL, USA), resulting blots were blocked for 1 h at room temperature (RT) in 5% nonfat dry milk or, for phosphorylated proteins, in 5% BSA in 1X Tris‐buffered saline supplemented with Tween‐20 (TBS‐T), then incubated with primary antibody overnight at 4 °C. The following antibodies were used NCS1 (ab116230; Abcam, Cambridge, MA, USA; diluted 1 : 2000), GAPDH (Cell Signaling Technology, Danvers, MA, USA, #2118; diluted 1 : 5000), Akt (Cell Signaling #9272; diluted 1 : 1000), phospho‐Akt (Ser473; Cell Signaling #9271; diluted 1 : 1000), NFκB p65 (Abcam ab16502; diluted 1 : 2000), nuclear factor of kappa‐light polypeptide gene enhancer in B‐cell inhibition, alpha (IκBα; Abcam ab7217; diluted 1 : 500), Lamin (Abcam ab8984; diluted 1 : 500). Following primary antibody incubation, membranes were washed with 1X TBS‐T and incubated with a horseradish peroxidase‐labeled goat IgG (Santa Cruz Biotechnology) at RT for 2 h. Membranes were washed, incubated in Pierce ECL western blotting solution (Thermo Fisher Scientific) and developed on X‐ray film in a dark room.

### 2.5 TRANSFAC database analysis

The TRANSFAC database (Matys *et al.*, 2006) was used to identify potential transcription factor binding sites in the predicted human *NCS1* promoter region. After obtaining a list of transcription factors that potentially bind 200 kB upstream to 200 kB downstream of the predicted transcriptional start site (TSS) of *NCS1,* we reviewed the literature on their involvement in Ca^2+^ signaling or cancer progression.

### 2.6 Assessment of TNF signaling *via* NFκB and NCS1 expression in breast cancer RNA microarray dataset

RNA microarray analysis was performed on normal human breast (*n* = 5) and human breast tumor (*n* = 8) tissue as described previously (Harvell *et al.*, 2013). Data were obtained from the Gene Expression Omnibus (http://www.ncbi.nlm.nih.gov/geo/query/acc.cgi?acc=GSE31192) and analyzed using Qlucore Omics Explorer (Lund, Sweden). Gene expression values were log_2_‐transformed and fold change calculated. A curated list of genes associated with TNF signaling *via* NFκB (BioCarta Gene Set M5890) was collected from Gene Set Enrichment Analysis Gene Sets (Broad Institute) and used to generate an expression heat map of differentially regulated (*P* < 0.05) NFκB‐regulated genes in normal versus cancerous breast tissue. Gene expression values of *NCS1* mRNA were plotted in normal versus cancerous breast tissue and against expression values of the catalytic subunit of protein phosphatase 2A (*PP2Ac*).

### 2.7 Alteration of NCS1 levels in MDA‐MB231 cells

To study the cellular effect of high NCS1 expression, MDA‐MB231 cells were stably transfected with a vector encoding for human *NCS1* or a control vector as described previously (Moore *et al.*, 2017). Breast cancer cells lacking NCS1 expression (NCS1 KO) were generated through CRISPR/Cas9 gene editing. The CRISPR/Cas9 vector used, pSpCas9(BB)‐2A‐GFP (PX458), was a gift from Feng Zhang (Addgene plasmid #48138, Watertown, MA, USA) (Ran *et al.*, 2013). Control cells (WT) were transfected with a PX458 vector containing a scrambled gRNA sequence. To generate NCS1 KO cells, the CRISPR/Cas9 vector was modified to contain gRNA targeting *NCS1*. For this purpose, the following gRNA pair was designed: forward, 5′‐CACCGTTGAAGCCCGAAGTTGTGG‐3′ reverse, 5′‐AAACCCACAACTTCGGGCTTCAAC‐3′. After annealing these two nucleotide sequences, the gRNA was inserted in the PX458 vector backbone. The construct was transformed into DH5α bacteria, selected with ampicillin, and isolated using a QIAprep Spin Miniprep Kit (QIAGEN) according to the manufacturer's protocol. The insert was validated by sequencing. Quality and concentration of the DNA were validated with spectrophotometry (NanoDrop; Thermo Scientific). For transfection of MDA‐MB231 cells, MDA‐MB231 cells were plated on 100‐cm dishes 24 h prior to transfection. Afterward, cells were transfected for 48 h with either the NCS1‐PX458 vector or the scrambled PX458 vector control. Transfection was performed using FuGene HD transfection reagent (Promega, Madison, WI, USA) according to the manufacturer's protocol. As the PX458 vector contains GFP, FACS cell sorting was performed to isolate successfully transfected cells by selecting single GFP‐positive cells into 96‐well plates. These cells were grown in complete growth medium until the wells were confluent. Colonies were tested for successful NCS1 KO by western blot analysis and IF. The primary antibody for NCS1 was obtained from Abcam (ab116230) and was used in 1 : 2000 dilution for western blotting and 1 : 500 for IF.

### 2.8 Fluorescence microscopy

For fluorescence microscopy, MDA‐MB231 WT and NCS1 KO cells were seeded on sterile glass coverslips and grown to 80% confluency. Medium was removed, and each coverslip was washed twice with 2 mL of 1× PBS (pH 7.4; American Bio, Natick, MA, USA). Fixation was performed for 15 mi at RT with 4% paraformaldehyde. After several washes in 1× PBS supplemented with 0.1% Tween‐20 (PBS‐T), cells were permeabilized in 0.1% Triton X‐100 for 5 min. Following permeabilization, cells were washed and blocked for 1 h at RT in 1X PBS‐T supplemented with 10% normal goat serum (Cell Signaling Technology Inc.). Cells were incubated at 4 °C overnight with a rabbit anti–NCS1 monoclonal antibody (ab116230; Abcam; diluted 1 : 500) diluted in blocking solution. After washing with PBS, cells were incubated with AlexaFluor‐488 goat anti‐rabbit secondary antibody (1 : 1000 dilution; Thermo Fisher Scientific) and rhodamine‐conjugated phalloidin (1 : 1000 dilution; Thermo Fisher Scientific) for 2 h at RT in the dark. Cells were washed with PBS and mounted on glass slides with ProLong Gold Antifade Mountant with DAPI (Thermo Fisher Scientific). Slides were cured overnight before images were captured with a confocal microscope (LSM 710 Duo; Carl Zeiss, Oberkochen, Germany).

### 2.9 Proliferation assay

Cell proliferation was assessed using a luminescent‐based assay that measures the number of viable cells in culture based on ATP levels (CellTiter‐Glo Luminescence Cell Viability Assay [Promega]). One thousand cells were seeded into each well of a sterile 96‐well plate except for the wells at the edges of the plate. The relative number of viable cells was measured from day 0 to 4 by adding 100 μL of CellTiter‐Glo Reagent (Promega) to 10 wells per timepoint and genotype. The cells were incubated in this reagent for 15 min at RT to stabilize the luminescent signal. Luminescence was measured on a microplate reader (Tecan Infinite M1000 Pro; Tecan Trading, Mannedorf, Switzerland). Three independent experiments were performed using MDA‐MB231 WT and NCS1 KO cells with 10 wells per timepoint and genotype.

### 2.10 Scratch motility assay

Scratch motility assays were performed by growing MDA‐MB231 WT and NCS1 KO cells to confluency in six‐well plates. After cells were serum‐starved (1% FBS) for 12 h, a T‐shaped wound was induced in the cell layer using a sterile pipette tip. Cells were washed with sterile PBS to remove detached cells. Wound width was assessed at timepoints of 0 and 24 h by capturing images of the scratch under a light microscope. Wound widths at different locations of the wound were measured using ImageJ software and the mean for each wound was calculated. The difference between the width at 0 and 24 h was calculated and depicted as mean wound closure in cm in 24 h. Four independent experiments were performed in triplicate for each genotype.

### 2.11 Colony formation assay

To study cell survival, we performed colony formation assays on MDA‐MB231 WT and NCS1 KO cells. MDA‐MB231 WT or NCS1 KO cells were seeded on 12‐well plates at an initial number of 100 or 500 cells. Cells were counted using a hematocytometer and incubated for 10 days in complete growth medium at 37 °C and 5% CO_2_. After 10 days, cells were fixed with 100% ice‐cold methanol and colonies were stained with 2.5% crystal violet. Plates were scanned with a conventional scanner and the area covered by cell colonies was analyzed with ImageJ (Guzmán *et al.*, 2014). Data were obtained from 4 independent experiments with four replicates in each experiment and expressed as area covered with colonies in %.

### 2.12 Calcium imaging

Fluorescence‐based Ca^2+^ imaging was performed using a fluorescence microscope (Orca 2 camera imaging system [Metamorph] mounted on a Zeiss inverted microscope). To measure the response to UTP, the Ca^2+^ reporter dye Fluo‐4‐AM (Thermo Fisher Scientific) was used. Baseline Ca^2+^ and response to TG were measured using the ratiometric dye Fura‐2‐AM (Thermo Fisher Scientific). Ca^2+^ responses to UTP and TG were measured in Ca^2+^‐free conditions to abolish effects of extracellular Ca^2+^ on the measured response. Baseline Ca^2+^ was assessed in Ca^2+^‐containing conditions. Ca^2+^‐containing HEPES‐buffered saline (HBS) contained 1.25 mm CaCl_2_, 19.7 mm HEPES, 4.7 mm KCl, 1.2 mm KH_2_PO_4,_ 1 mm MgSO_4,_ 130 mm NaCl, and 5 mm dextrose in dH_2_O, pH 7.4 at RT. In Ca^2+^‐free HBS buffer CaCl_2_ was replaced by 1.25 mm MgCl_2_ and additionally 0.1 mm EGTA was added. MDA‐MB231 WT or NCS1 KO cells were plated on glass coverslips 1 day prior to imaging at a density of 100 000 cells per coverslip. After 1 day of growth, cells were incubated at 37 °C in 5% CO_2_ for 40 min with Fura‐2‐AM or Fluo‐4‐AM dissolved in Ca^2+^‐containing HBS buffer with 0.03% pluronic acid (Thermo Fisher Scientific). Excess dye was washed away with Ca^2+^‐containing HBS buffer. Cells were incubated for another 10 min in Ca^2+^‐containing HBS. To measure the effect of the intracellular Ca^2+^ chelator BAPTA‐AM on baseline Ca^2+^ levels, WT and NCS1 KO cells were incubated with Fura‐2‐AM supplemented with 1 μm BAPTA‐AM prior to imaging. Once in view on the fluorescence microscope, approximately 20 single cells plus one empty area (background) were identified and defined as regions of interest. For Ca^2+^ responses to UTP and TG, cells were perfused with Ca^2+^‐free HBS for 30 s before imaging was started. After 10 s of imaging (F0), cells were stimulated with 10 µm UTP or 5 µm TG in Ca^2+^‐free HBS. After 30 s of stimulation with UTP or 60 s stimulation with TG, cells were perfused with Ca^2+^‐free HBS until the fluorescence signal returned to baseline. For baseline Ca^2+^ measurements, the ratiometric fluorescence signal of unstimulated cells in Ca^2+^‐containing HBS buffer was recorded over 100 s. To investigate the Ca^2+^ response to stimulation with UTP measured with Fluo‐4‐AM, data were analyzed by subtracting the background from all measured values followed by normalization of each timepoint to the average of the first 10 s (F1/F0). Maximal amplitude, area under the curve (AUC), and rate of rise were calculated using graphpad prism 7 (San Diego, CA, USA). Baseline Ca^2+^ measurements with Fura‐2‐AM were analyzed by calculating the mean ratio of fluorescence at 340 and 380 nm under unstimulated conditions in Ca^2+^‐containing HBS buffer. The response to TG measured with Fura‐2‐AM was analyzed by normalizing the 340/380 ratio of each timepoint to the mean 340/380 ratio at baseline.

### 2.13 Statistical analysis

Unless otherwise specified, each experiment was performed at least three times. Depicted are bar graphs including individual data points showing mean ± SD or box plots showing the median, interquartile range, and whiskers ranging from the minimum to maximum value. Experiments were blinded for treatments and genotypes where applicable. Statistical analyses were performed using graphpad prism 7. Gaussian distribution of datasets was checked with Shapiro–Wilk normality test before applying parametric statistical tests. Significance between two groups was assessed with unpaired Student's *t*‐tests if the data passed normality test. For comparison of groups of three or more, ordinary one‐way ANOVA followed by Tukey *post hoc* test was performed. In cases of failed normality test, we applied nonparametric Mann–Whitney *U*‐test. *P* values < 0.05 were considered statistically significant. *P < 0.05, **P < 0.01, ***P < 0.001.

## 3 Results

### 3.1 NCS1 is up‐regulated in response to NFκB‐activating stressors

**Fig. 1:**
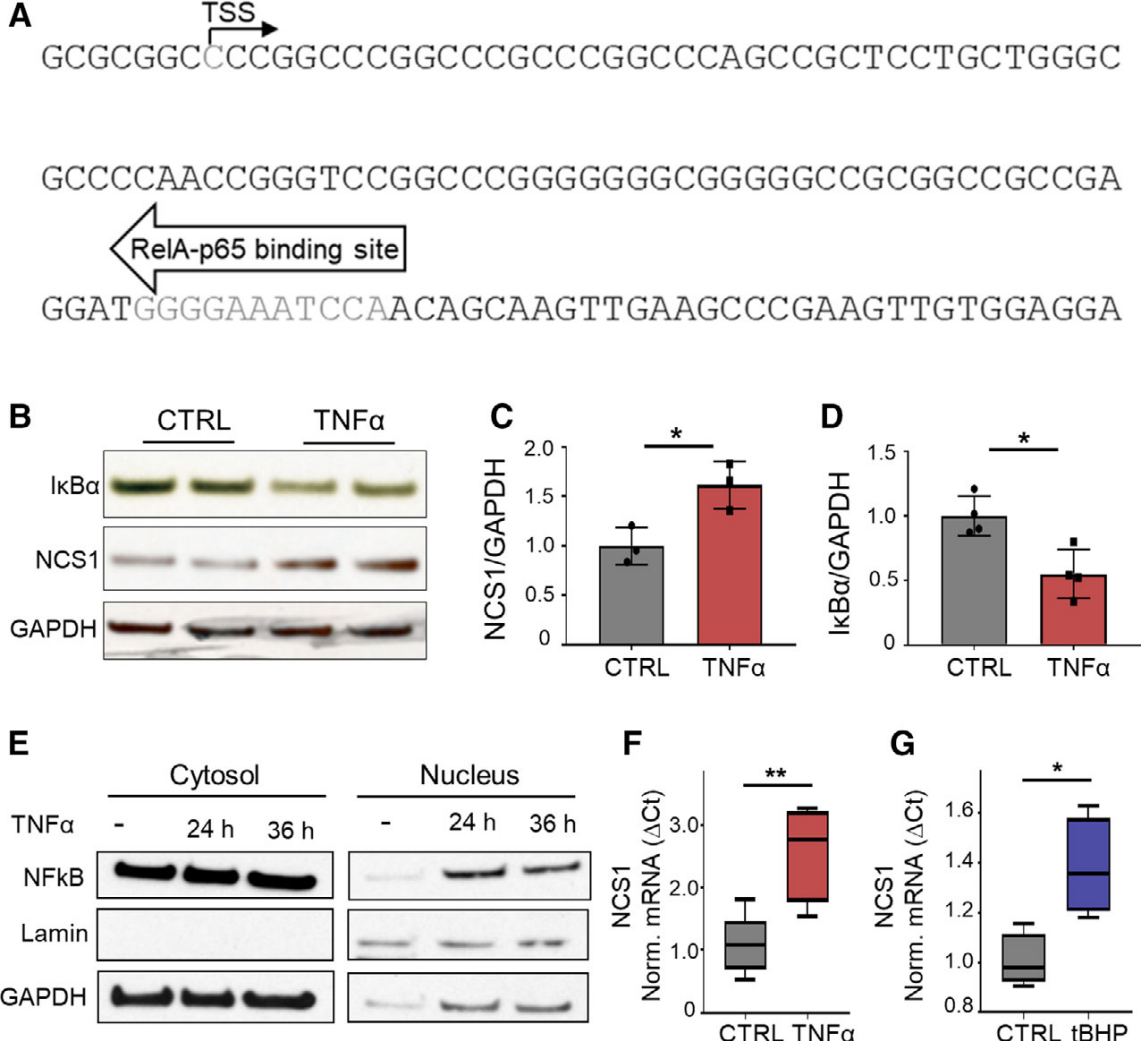
NCS1 is up‐regulated in response to NFκB‐activating stressors. (A) *NCS1* promoter region predicted by TRANSFAC (Matys *et al.*, 2006) with the TSS and binding site for the NFκB subunit RelA‐p65 located 88 kilobases downstream of the TSS. (B) Western blot analysis of SHSY5Y whole‐cell lysate after treatment with DMSO (CTRL) or 10 ng·mL^−1^ TNF‐α for 24 h. Depicted is a representative blot. (C) Quantification of NCS1 protein levels normalized to GAPDH shows that TNF‐α treatment significantly increased NCS1 protein levels. Depicted is the mean ± SD of *n* = 3 independent experiments. *P < 0.05, determined by unpaired Student's *t*‐test. (D) Quantification of IκBα protein levels normalized to GAPDH shows that TNF‐α treatment significantly decreased IκBα protein. Depicted is the mean ± SD of *n* = 4 independent experiments. *P < 0.05, determined by unpaired Student's *t*‐test. (E) Representative western blot of cytosolic and nuclear fractions from SHSY5Y cells after 24 h of treatment with 10 ng·mL^−1^ TNF‐α showing nuclear translocation of transcription factor NFκB after TNF‐α treatment. (F, G) Quantitative real‐time PCR of SHSY5Y cells treated with NFκB‐activating stimuli or DMSO control (CTRL). (F) *NCS1* mRNA increase after treatment with 10 ng·mL^−1^ TNF‐α for 24 h. Depicted are box plots of *n* = 4 independent experiments. **P < 0.01, determined by unpaired Student's *t*‐test. (G) *NCS1* mRNA increase after 20 h treatment with 1 µm tBHP. Depicted are box plots of *n* = 4 biological replicates. *P < 0.05, determined by unpaired Student's *t*‐test.

To identify stimuli that lead to altered NCS1 expression, we investigated transcription factors predicted to regulate NCS1 gene transcription. For this purpose, we analyzed the predicted promoter region of *NCS1* to identify potential transcription factor binding sites using TRANSFAC, a database encompassing information on eukaryotic transcription factors, their genomic binding sites, and DNA binding profiles (Matys *et al.*, 2006). Our analysis revealed a binding site for the NFκB subunit RelA‐p65 within the predicted *NCS1* promoter, 88 to 99 kilobases downstream of the predicted *NCS1* TSS (Fig. 1A). TNF‐α is a well‐established activator of the transcription factor and regulator of cellular stress, NFκB (Balkwill, 2009; Ben‐Neriah and Karin, 2011; Oliveira‐Marques *et al.*, 2007; Pahl, 1999). TNF‐α binds to TNF receptors 1 and 2 to activate the IκBα kinase complex, leading to the degradation of IκBα and consequent nuclear translocation of NFκB. In the nucleus, NFκB binds to specific DNA binding motifs to regulate the expression of a variety of different genes (Pahl, 1999; Tian *et al.*, 2005). To test whether NCS1 is regulated by NFκB, we treated SHSY5Y human neuroblastoma cells with TNF‐α and consequently measured NCS1 protein expression. NCS1 protein abundance increased in SHSY5Y cells treated with TNF‐α for 24 h (Fig. 1B,C). NFκB activation after TNF‐α treatment was confirmed by immunoblotting for IκBα in whole‐cell lysate and for NFκB in cytosolic and nuclear cell fractions. Treatment with TNF‐α for 24 h resulted in IκBα degradation (Fig. 1B,D) and translocation of NFκB to the nucleus (Fig. 1E), confirming NFkB activation. Measuring *NCS1* mRNA expression showed increased mRNA levels of *NCS1* following treatment with TNF‐α (Fig. 1F), suggesting that NCS1 up‐regulation happens on a transcriptional level. To induce NFκB signaling through an additional stressor, we treated SHSY5Y cells for 20 h with tBHP, which was also reported to activate NFκB (Pahl, 1999). Measurements of *NCS1* mRNA expression again showed an increase of *NCS1* following treatment with tBHP (Fig. 1G). Other conditions, namely high extracellular Ca^2+^ or TG, did not change *NCS1* expression (Fig. S1). These results suggest that NCS1 is transcriptionally up‐regulated by extracellular stressors that specifically activate NFκB.

### 3.2 TNF‐α‐induced NCS1 up‐regulation is NFκB‐dependent

**Fig. 2:**
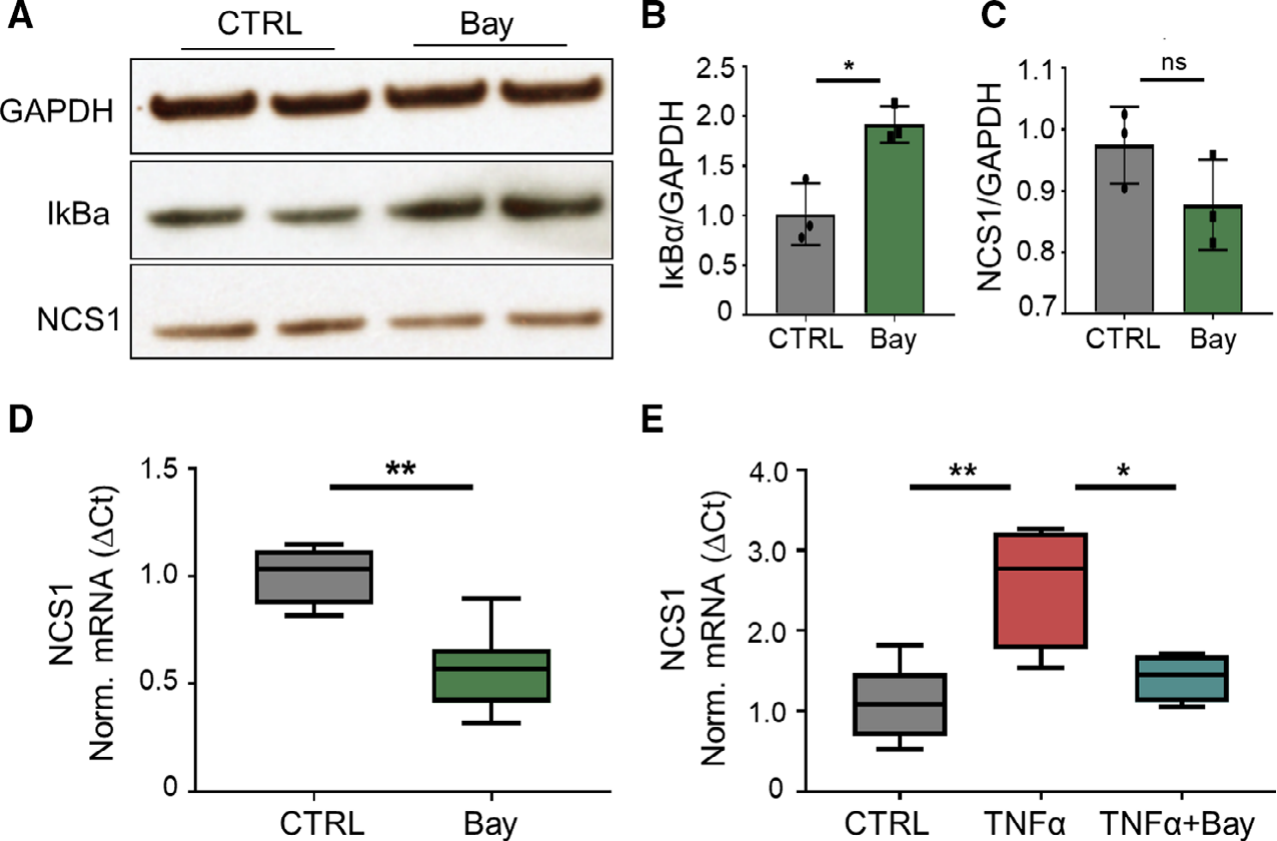
TNF‐α‐induced NCS1 up‐regulation is NFκB‐dependent. (A) Western blot analysis of SHSY5Y whole‐cell lysates treated with DMSO (CTRL) or 1 µm of the NFκB inhibitor Bay 11‐7082 (Bay) for 24 h. Depicted is a representative blot. Bay treatment significantly increased IκBα compared to CTRL as quantified in (B). Depicted is the mean ± SD of *n* = 3 independent experiments, *P < 0.05, determined by unpaired Student's *t*‐test. (C) 24 h of NFκB inhibition caused a slight, but not significant decrease of NCS1 protein. Depicted is the mean ± SD of *n* = 3 independent experiments, *P* > 0.05, determined by unpaired Student's *t*‐test. (D) Quantitative real‐time PCR of SHSY5Y cells treated with DMSO (CTRL) or 1 µm Bay for 24 h. *NCS1* mRNA expression decreased with NFκB inhibition compared to CTRL. Depicted are box plots of *n* = 4 independent experiments. **P < 0.01, determined by unpaired Student's *t*‐test. (E) Quantitative real‐time PCR of SHSY5Y treated with DMSO (CTRL), 10 ng·μL^−1^ TNF‐α, or 10 ng·μL^−1^ TNF‐α + 1 µm Bay for 24 h. TNF‐α‐induced increase of *NCS1* mRNA (**P < 0.01) was prevented by cotreatment with Bay (*P < 0.05). Depicted are box plots of *n* = 4 independent experiments. Statistical significance was determined by ordinary one‐way ANOVA followed by Tukey *post hoc* test.

To conclusively determine whether TNF‐α‐induced NCS1 up‐regulation is caused by NFκB activation, we examined the effect of pharmacological NFκB inhibition on NCS1 expression using the NFκB inhibitor Bay 11‐7082 (Bay). This compound inhibits the degradation of IκBα, therefore preventing nuclear translocation of NFκB. After treating SHSY5Y cells with Bay for 24 h and immunoblotting for IκBα, we saw that IκBα protein levels increased (Fig. 2A,B), demonstrating the successful inhibition of IκBα degradation and hence NFκB activation. Treatment with the NFκB inhibitor alone caused a slight but not significant decrease of NCS1 protein levels (Fig. 2A,C), but did significantly decrease *NCS1* mRNA expression (Fig. 2D). These data suggest that NFκB regulates transcription of *NCS1,* but NCS1 protein has a longer half‐life than our treatment time of 24 h. We hypothesize that longer treatment with Bay would show diminished levels of NCS1 protein. Cotreatment of cells with TNF‐α and Bay abrogated the TNF‐α‐induced NCS1 increase (Fig. 2E), confirming that TNF‐α induces *NCS1* expression through a NFκB‐dependent mechanism.

### 3.3 NCS1 up‐regulation corresponds with NFκB activation in human breast cancer

**Fig. 3:**
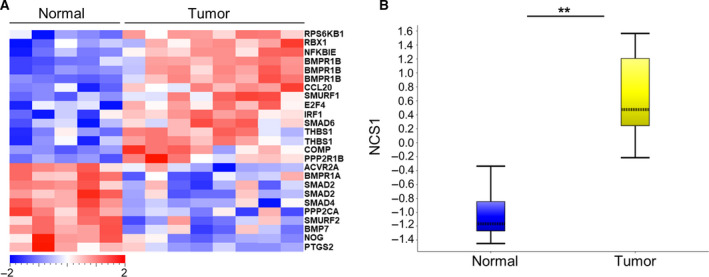
NCS1 and NFκB‐regulated genes are differentially expressed in human breast cancer tissue compared to normal breast tissue. (A) Expression heat map of differentially expressed (*P* < 0.05) NFκB‐regulated genes in normal (*n* = 5) versus cancerous breast tissue (*n* = 8). NFκB‐related genes show distinct expression patterns between groups, indicating altered NFκB‐signaling in human breast cancer. A color legend is pictured with a scale from −2 to + 2‐fold change. (B) Gene expression values of *NCS1* mRNA were plotted in normal (*n* = 5) versus cancerous breast tissue (*n* = 8) and revealed increased NCS1 gene expression in human breast cancer compared to normal breast tissue. **P < 0.01, determined by Student's *t*‐test. RNA microarray analysis was performed as described previously (Harvell *et al.*, 2013). Data were obtained from the Gene Expression Omnibus (http://www.ncbi.nlm.nih.gov/geo/query/acc.cgi?acc=GSE31192).

To investigate the relevance of TNF signaling *via* NFκB activation and NCS1 up‐regulation in human cancer, we analyzed the gene expression patterns of NFκB‐regulated genes and *NCS1* expression in a dataset from an RNA microarray performed on human breast cancer and noncancerous breast tissue. A heat map of NFκB‐regulated genes was generated from RNA expression values of noncancerous (normal) and cancerous (tumor) breast cancer. We found that genes transcriptionally regulated by NFκB have a distinct expression pattern in breast cancer tissue compared to normal breast tissue, indicating the differential regulation of NFκB signaling in breast cancer (Fig. 3A). Furthermore, we found that *NCS1* expression was significantly higher in breast cancer samples compared to normal breast samples (Fig. 3B). These findings give relevance to the results from our *in vitro* experiments, as they show that *NCS1* up‐regulation corresponds with NFκB activation in human breast cancer pathophysiology. In addition, these results further support our hypothesis that NCS1‐dependent signaling has a specific function in breast cancer.

### 3.4 CRISPR/Cas9 knockout of NCS1

**Fig. 4:**
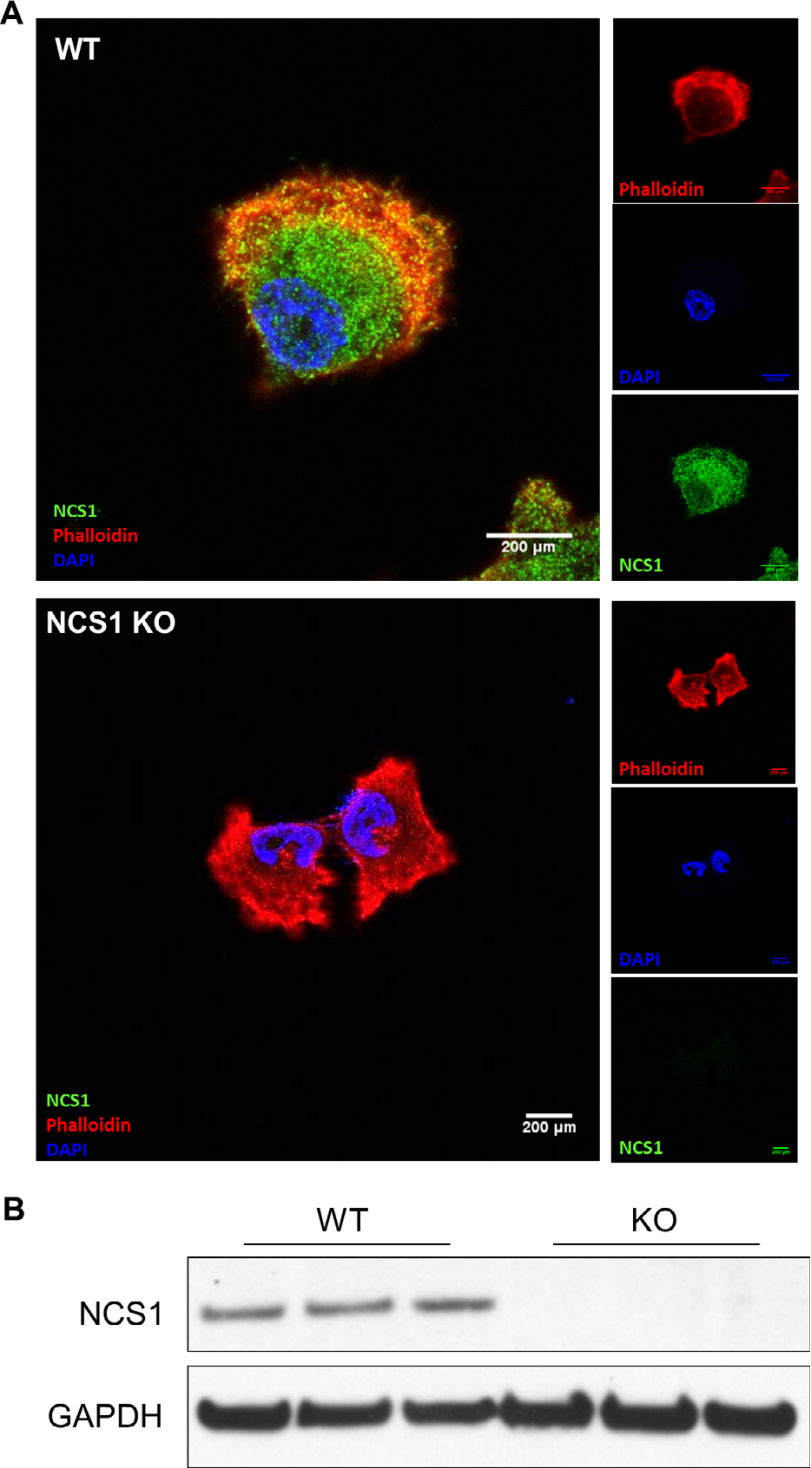
Generation of NCS1 KO MDA‐MB231 cells. (A) Representative IF images of WT and NCS1 KO MDA‐MB231 cells stained for NCS1 (green), phalloidin (red), and DAPI (blue). The large panels show merged images of all three channels and the small panels show the different channels separately. (B) Western blot of WT and NCS1 KO MDA‐MB231 cells, confirming successful deletion of NCS1 protein expression in KO cells.

Having determined that NFκB signaling and NCS1 are altered in human breast cancer, we moved to investigate the specific role of NCS1 in breast cancer. To this end, we generated MDA‐MB231 cells lacking NCS1 using CRISPR/Cas9 technology. MDA‐MB231 NCS1 KO and control (WT) cells were confirmed through immunofluorescence (IF) staining (Fig. 4A) and western blot analysis (Fig. 4B).

### 3.5 NCS1 KO renders cells deficient in colony formation and migration capabilities

**Fig. 5:**
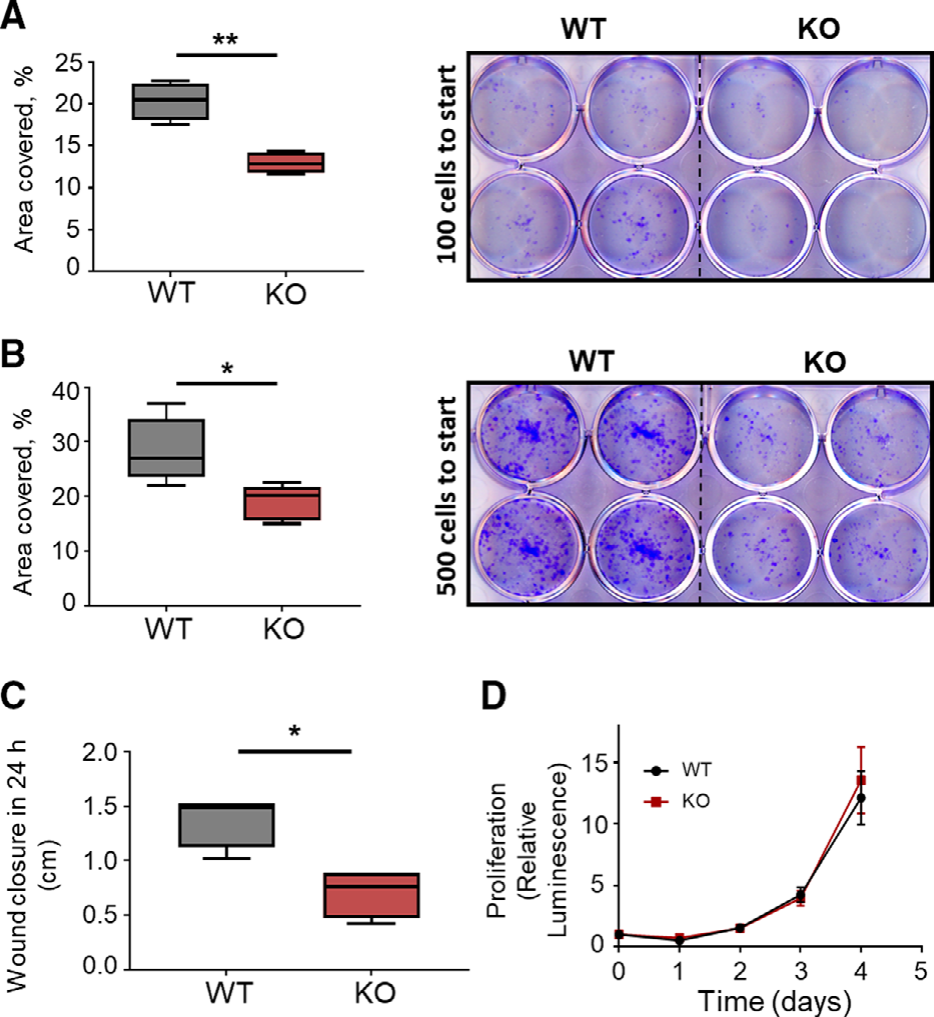
NCS1 KO reduces cell survival and motility. (A, B) Colony formation assays of WT and NCS1 KO MDA‐MB231 cells. Cells were seeded at a density of (A) 100 cells per well or (B) 500 cells per well. After 10 days in culture, the culture area covered by colonies was analyzed with ImageJ, demonstrating that NCS1 KO cells had a diminished capacity to grow colonies and survive compared to WT cells (the area covered with colonies is shown as % total area). Depicted are box plots of *n* = 4 independent experiments. **P < 0.01 (A) and *P < 0.05 (B), determined by unpaired Student's *t*‐test. (C) Scratch assay demonstrating the wound healing capacity of MDA‐MB231 WT and NCS1 KO cells. Depicted is the mean difference between the wound width at 0 and 24 h, that is, the wound closure in 24 h in cm following wound induction. NCS1 KO cells moved significantly less compared to WT cells. The wound width was measured with ImageJ, and the wound closure is expressed in cm. Depicted are box plots of *n* = 4 independent experiments. *P < 0.05, by Mann–Whitney *U*‐test. (D) Proliferation assay demonstrating no difference in cell proliferation between NCS1 KO and WT cells. Cell proliferation was assessed with CellTiter‐Glo Luminescence Cell Viability Assay (Promega) at different timepoints (0–4 days) after plating 1000 cells per well on a 96‐well plate. Depicted is the relative Luminescence compared to timepoint 0. Each timepoint shows the mean luminescence ± SD (*n* = 10).

Whereas previous publications focused on the effect of NCS1 overexpression (NCS1 OE) on promoting tumor aggressiveness (Apasu *et al.*, 2019; Moore *et al.*, 2017), here we investigate the effect of NCS1 KO on cell survival, migration, and proliferation. Colony formation assays in MDA‐MB231 WT and NCS1 KO cells were used to study cell survival. Assays were performed with 100 or 500 cells seeded in six‐well cell culture dishes and cells were allowed to grow undisturbed for 10 days. After fixing and staining the resulting colonies, the percentage of culture dish area covered by colonies was calculated. NCS1 KO formed significantly fewer colonies compared to WT cells (Fig. 5A,B) suggesting poorer cell survival. We then measured the effect of NCS1 on cell migration by performing wound healing assays on WT and NCS1 KO cells. After a standardized wound was induced in WT and NCS1 KO cells growing in culture, wound closure was quantified by measuring the distance that cells moved in 24 h. NCS1 KO moved significantly less compared to WT (Fig. 5C, Fig. S2A–D), demonstrating defective migration and survival capabilities in these cells. Cell proliferation was then assessed using a luminescence‐based assay that measures the number of viable cells based on the level of ATP present, but no significant difference in proliferation was seen between WT and NCS1 KO cells (Fig. 5D), consistent with previous findings (Moore *et al.*, 2017; Wang *et al.*, 2016). These results demonstrate an important role for NCS1 in cell survival and migration, but not cell proliferation.

### 3.6 NCS1 knockout reduces InsP3‐dependent ER Ca^2+^ release and increases baseline cytosolic Ca^2+^ levels, which can be rescued by intracellular Ca^2+^ chelation

**Fig. 6:**
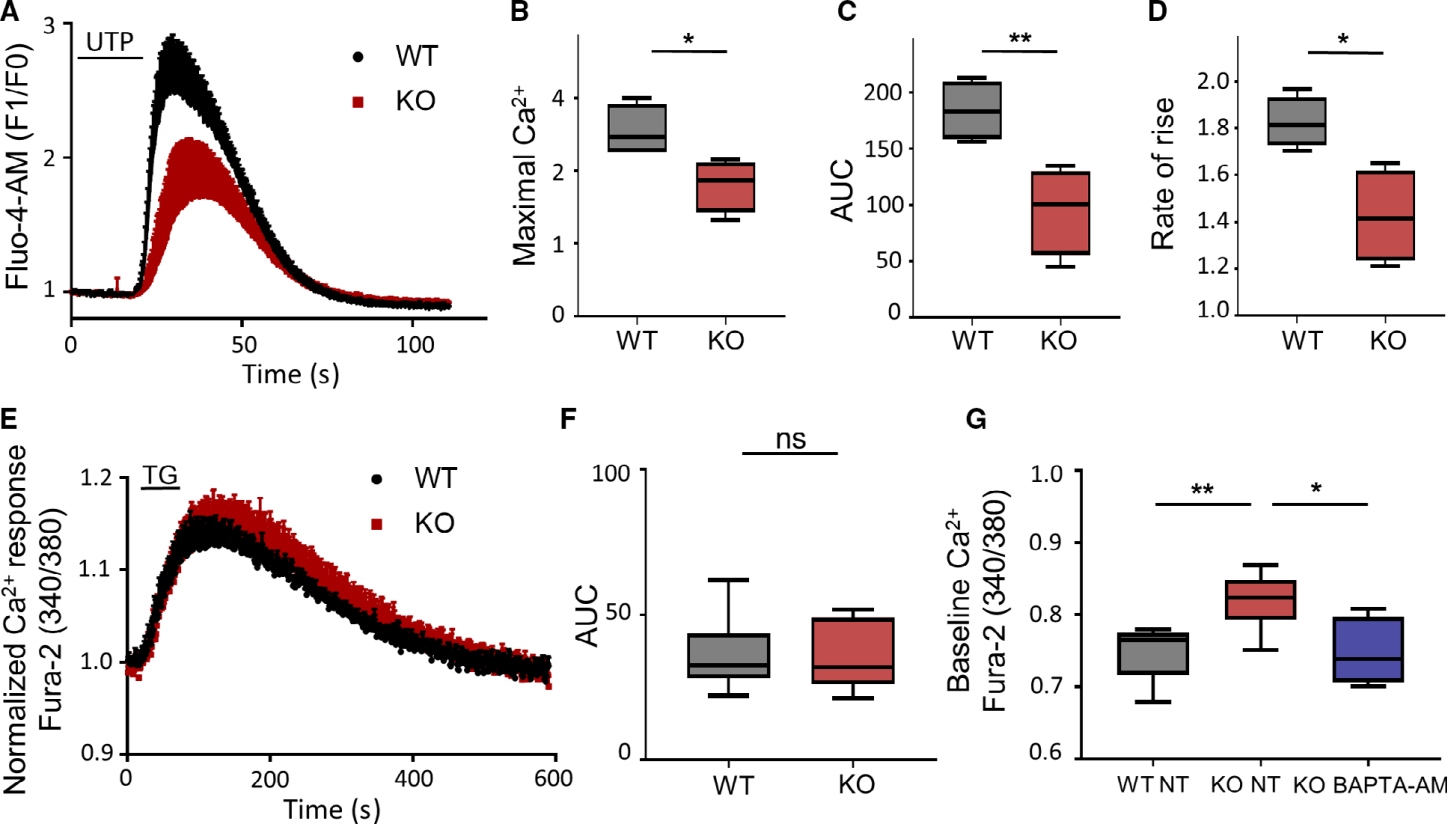
NCS1‐deficient cells exhibit reduced InsP3‐dependent Ca^2+^ responses and have increased cytosolic Ca^2+^ levels at baseline (A) InsP3‐dependent Ca^2+^ response of WT and NCS1 KO MDA‐MB231 cells. Ten micromolar UTP was used to stimulate Ca^2+^ release from internal stores. Depicted are the Ca^2+^ responses to a 20 s stimulation with UTP in Ca^2+^‐free conditions, shown as the fluorescence signal of Fluo‐4‐AM at timepoint × (F1) compared to the baseline fluorescence (F0). Each timepoint shows the mean ± SD of four coverslips for each genotype. (B) Box plots showing the maximal amplitude of the Ca^2+^ response depicted in (A), demonstrating a significantly smaller amplitude in NCS1 KO compared to WT cells. *P < 0.05, determined by unpaired Student's *t*‐test. (C) Box plots depicting the AUC from the experiment shown in (A), demonstrating a significantly smaller AUC in NCS1 KO compared to WT cells. **P < 0.01, determined by unpaired Students *t*‐test. (D) Box plots depicting the Ca^2+^ transient rate of rise from the experiment shown in A, demonstrating a significantly smaller Ca^2+^ transient rate of rise in NCS1 KO cells than in WT cells. *P < 0.05, determined by unpaired Student's *t*‐test. (E) Depletion of ER Ca^2+^ stores of WT and NCS1 KO MDA‐MB231 cells induced by TG. Depicted are the Ca^2+^ responses following stimulation with 5 μm TG (starting at 10 s for 60 s) in Ca^2+^‐free conditions shown as 340/380 ratio normalized to the 340/380 ratio before applying TG measured with the Ca^2+^ indicator Fura‐2‐AM. Each timepoint shows the mean ± SD of nine coverslips for each genotype. (F) Box plots showing the AUC from the experiment shown in (E). No significant difference in the Ca^2+^ response to TG of NCS1 KO compared to WT cells could be revealed. Determined not significant (ns) by Mann–Whitney *U*‐test. (G) Box plots showing baseline Ca^2+^ levels of WT and NCS1 KO MDA‐MB231 cells measured with the Ca^2+^ indicator Fura‐2‐AM in Ca^2+^‐containing conditions and shown as 340/380 ratio. NCS1 KO cells have significantly higher cytosolic Ca^2+^ levels at baseline compared to WT cells (**P < 0.01). Pretreatment of NCS1 KO cells with 1 μm of the intracellular Ca^2+^ chelator BAPTA‐AM for 30 min rescued the increased baseline Ca^2+^ levels of NCS1 KO cells and reduced them almost to levels of NCS1 WT cells (*P < 0.05). No difference between WT NT and KO BAPTA‐AM could be observed (*P* > 0.05). *N* = 5–9 coverslips for each condition. Significance was assessed by ordinary one‐way ANOVA followed by Tukey *post hoc* test.

Neuronal calcium sensor 1 is an established Ca^2+^‐binding protein (Burgoyne, 2007), and it is known that NCS1 increases InsP3R activity at the single channel level (Schlecker *et al.*, 2006). Because the InsP3R is known to regulate cancer progression (Akl and Bultynck, 2013), we aimed to determine how NCS1 affects cellular Ca^2+^ homeostasis and signaling in breast cancer cells. For this purpose, we performed fluorescence imaging of intracellular Ca^2+^ in WT and NCS1 KO MDA‐MB231 cells to investigate NCS1‐dependent regulation of InsP3‐dependent ER Ca^2+^ release and baseline intracellular Ca^2+^ levels. The effect of NCS1 on InsP3‐dependent Ca^2+^ responses in WT and NCS1 KO cells was monitored using fluorescence imaging of Ca^2+^ transients in live cells stimulated with UTP. UTP is an agonist of the purinergic P2Y receptor, causing phosphoinositide phospholipase C‐mediated InsP3 generation and consequent Ca^2+^ release from the ER through the InsP3R (Erb and Weisman, 2012). Imaging revealed that the amplitude of the Ca^2+^ response was decreased in NCS1 KO compared to WT cells (Fig. 6A,B), and quantification of the AUC showed that the total amount of Ca^2+^ released was significantly less in NCS1 KO cells (Fig. 6C). Additionally, the Ca^2+^ transient rate of rise in NCS1 KO cells was significantly smaller than in WT cells (Fig. 6D). To rule out the possibility that the decreased Ca^2+^ response in NCS1 KO cells was due to decreased steady‐state ER Ca^2+^ content, we investigated the Ca^2+^ response to TG, a drug that induces ER Ca^2+^ depletion *via* inhibition of sarco/ER Ca^2+^‐ATPase. Stimulation of WT and NCS1 KO cells with 5 μm TG did not reveal any difference of the ER Ca^2+^ content between the two genotypes (Fig. 6E,F). To focus on Ca^2+^ responses from internal stores, the experiments with UTP and TG were performed in Ca^2+^‐free HBS. At baseline, we found a significantly higher fluorescence signal in NCS1 KO cells compared to WT cells, indicating increased resting Ca^2+^ levels with NCS1 KO. The increased baseline intracellular Ca^2+^ levels could be rescued by treatment of NCS1 KO cells with the intracellular Ca^2+^ chelator, BAPTA‐AM (Fig. 6G). These results show that upon InsP3‐generating stimuli, NCS1 enhances ER Ca^2+^ release independent of ER Ca^2+^ content and that under resting conditions, NCS1 is crucial for maintaining low intracellular Ca^2+^ levels through its function as a Ca^2+^ buffer.

### 3.7 NCS1 increases Akt activity

**Fig. 7:**
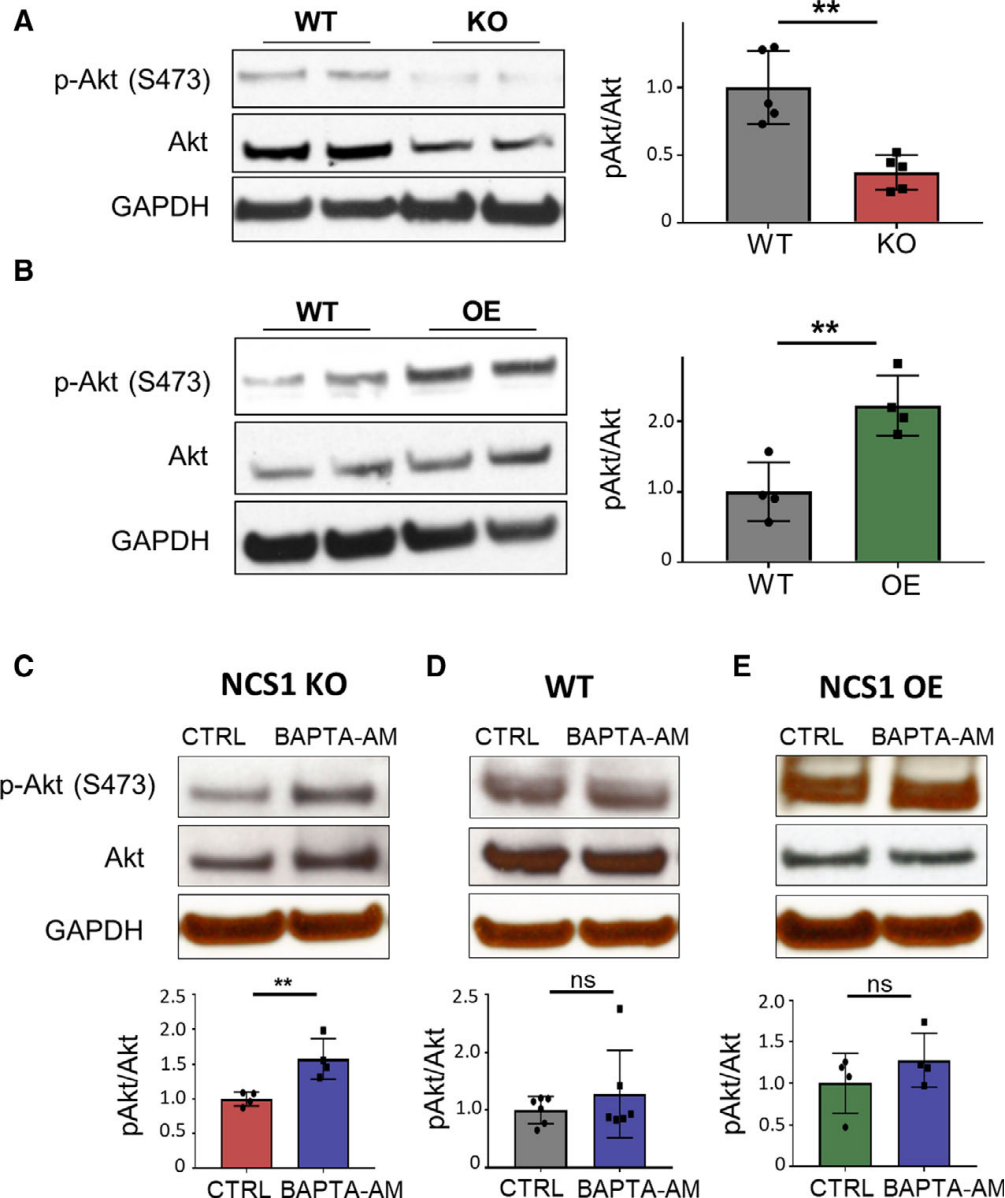
Impaired Akt signaling in NCS1 KO and NCS1 OE cells. (A) Western blot analysis of MDA‐MB231 WT and NCS1 KO cells showing decreased basal p‐Akt in NCS1 KO compared to WT. Depicted is a representative blot (left). **P < 0.01, determined by unpaired Student's *t*‐test (right). (B) Western blot analysis of WT and NCS1 OE MDA‐MB231 cells showing increased p‐Akt in NCS1 OE compared to WT. Depicted is a representative blot (left). **P < 0.01, determined by unpaired Student's *t*‐test (right). (C–E) Akt activation following intracellular Ca^2+^ buffering with BAPTA‐AM. Depicted are western blot analyses for total Akt (Akt) and p‐Akt in NCS1 KO (C), WT (D), and NCS1 OE (E) MDA‐MB231 cells after treatment with DMSO (CTRL) or 1 µm BAPTA‐AM for 30 min to buffer intracellular free Ca^2+^. (C) Buffering of intracellular Ca^2+^ significantly increased Akt phosphorylation in NCS1 KO cells. *N* = 4 independent experiments. **P < 0.01, determined by unpaired Student's *t*‐test. (D, E) Buffering of intracellular Ca^2+^ caused only a slight and not significant increase of Akt phosphorylation in WT (D) and OE (E) cells. *N* = 6 and *n* = 4 independent experiments, respectively. Determined not significant (ns) by Mann–Whitney *U*‐test. Depicted are representative blots. All data are shown as mean ± SD.

Akt, also known as protein kinase B (PKB), is a well‐established survival‐promoting protein (Song *et al.*, 2005) that also promotes cell migration and other cancer‐related processes (Chin and Toker, 2009; Xue and Hemmings, 2013). Akt has been linked to NCS1 signaling, as cardiomyocytes lacking NCS1 expression have less stress‐induced activation of the Akt pathway (Nakamura *et al.*, 2016). Here, we used MDA‐MB231 cells to investigate the effect of NCS1 on baseline Akt activity, comparing cells with WT, KO, and OE levels of NCS1 (Fig S3). Akt is activated through PI3K and phosphatidylinositol (3,4,5)‐trisphosphate, leading to phosphorylation and consequent activation of Akt at Threonine308 (T308) and Serine473 (S473). As phosphorylation at S473 indicates full Akt activation (Sarbassov *et al.*, 2005), we examined Akt activity through immunoblotting for Akt phosphorylation at S473 (p‐Akt (S473)). Western blot analysis of MDA‐MB231 WT, NCS1 KO, and NCS1 OE cells revealed decreased basal Akt activity in NCS1 KO compared to WT cells (Fig. 7A). Conversely, NCS1 OE cells showed increased basal Akt activity (Fig. 7B). Because NCS1 KO cells had increased baseline Ca^2+^ levels which could be rescued with the chelation of intracellular Ca^2+^, we investigated the effect of BAPTA‐AM on Akt activity (Fig. 7C–E). Buffering intracellular Ca^2+^ with BAPTA‐AM led to a significant increase of p‐Akt levels in NCS1 KO cells (Fig. 7C), whereas no effect could be observed in WT and NCS1 OE cells (Fig. 7D,E). These data suggest that increased baseline Ca^2+^ in NCS1 KO cells causes decreased basal Akt activity and that NCS1 buffering of intracellular Ca^2+^ is crucial for maintaining proper Akt signaling.

## 4 Discussion

**Fig. 8:**
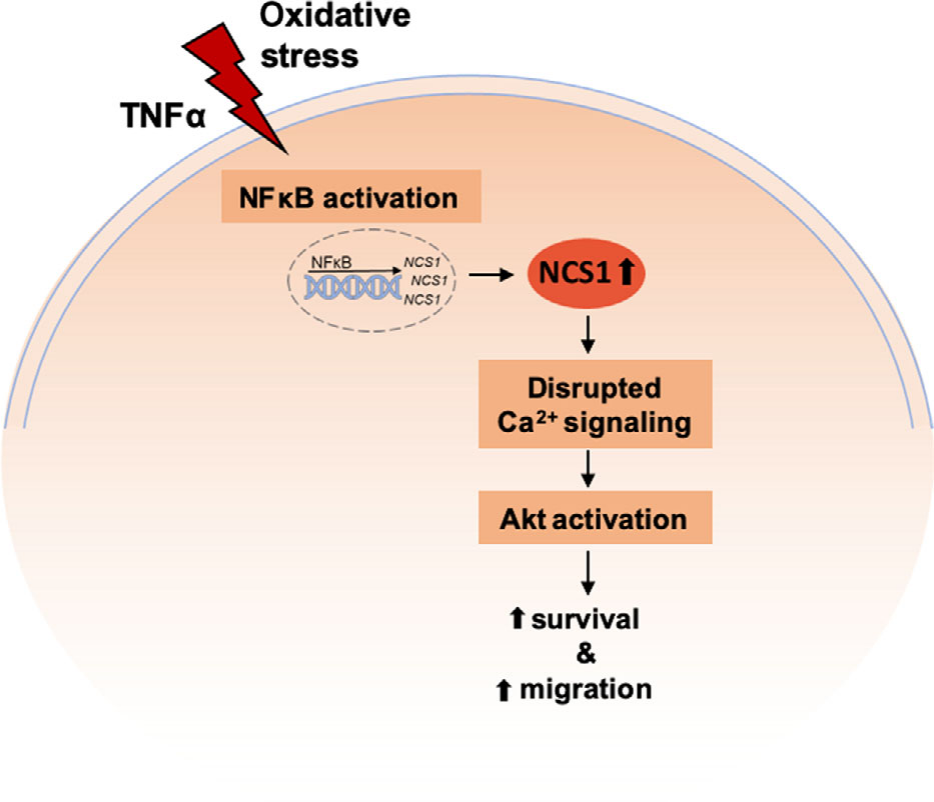
NCS1 as stress response protein: proposed mechanism. Environmental stressors such as TNF‐α or oxidative stress, which activate the transcription factor NFκB, lead to transcriptional up‐regulation of NCS1. NCS1 functions as cytosolic Ca^2+^ buffer, increases InsP3‐dependent ER Ca^2+^ release, and activates the Akt pathway. Increased NCS1 expression therefore causes disrupted Ca^2+^ signaling and increased Akt activity and consequently promotes cell survival and motility.

In this study, we (a) establish the Ca^2+^‐binding protein NCS1 as a stress response protein that is up‐regulated by NFκB‐activating stimuli, (b) reinforce its importance for cell survival and motility, and (c) demonstrate that NCS1 modulates Ca^2+^ signaling and Akt activity, both of which are important for cancer progression (Monteith *et al.*, 2017; Vivanco and Sawyers, 2002) (Fig. 8). A key finding of this study is that NCS1 is transcriptionally up‐regulated in response to cellular stress through the activation of NFκB. NFκB is a central regulator of the cellular stress response (Mercurio and Manning, 1999) and can be activated by a broad range of stimuli (Pahl, 1999). Cancer cells are embedded in a very complex microenvironment that consists of a variety of such environmental stressors, including TNF‐α (Cairns *et al.*, 2011; Landskron *et al.*, 2014; Salvatore *et al.*, 2017). NFκB is an established regulator of several pro‐survival and pro‐migratory genes (Baud and Karin, 2009; Ben‐Neriah and Karin, 2011; Hoesel and Schmid, 2013; Karin *et al.*, 2002; Taniguchi and Karin, 2018), and many types of cancer have been linked to constitutive NFκB activation (Baud and Karin, 2009; Karin *et al.*, 2002; Pires *et al.*, 2017; Piva *et al.*, 2006). We showed that treatment of SHSY5Y cells with the pro‐inflammatory cytokine TNF‐α activates the transcription factor NFκB and increases *NCS1* expression. Cotreatment with TNF‐α and an NFκB inhibitor prevented the up‐regulation of *NCS1*, indicating that TNF‐α‐induced up‐regulation of NCS1 is NFκB‐dependent. The oxidative stressor tBHP is also known to activate NFκB (Pahl, 1999) and led to up‐regulation of NCS1 as well. Additionally, analysis of the *NCS1* promoter region using TRANSFAC predicted a binding site for the NFκB subunit RelA‐p65 within the predicted promoter region of *NCS1*, further supporting our hypothesis that NCS1 is transcriptionally up‐regulated through NFκB‐activating signals. We demonstrated the relevance of these *in vitro* findings for human breast cancer through analysis of human RNA expression data, which showed the activation of NFκB signaling in breast cancer tissue in concordance with NCS1 up‐regulation. These findings indicate that up‐regulation of NCS1 *via* NFκB is relevant in human breast cancer pathophysiology. Although previous studies showed that high NCS1 expression leads to more aggressive behavior of tumor cells, specifically increased cell survival and motility and worse patient outcome (Apasu *et al.*, 2019; Moore *et al.*, 2017; Schuette *et al.*, 2018), our observations are a first step toward understanding how high NCS1 levels become elevated in cancer cells. Our results raise the possibility that targeting the NFκB‐activating tumor microenvironment could prevent up‐regulation of NCS1 and other stress response proteins that consequently lead to a more aggressive tumor phenotype.

With the evidence that there is a specific mechanism of NCS1 up‐regulation with stress, the question arises regarding the specific function of NCS1 in cancer cells, and which cellular signaling pathways are regulated by NCS1 to promote tumor aggressiveness. In the present study, we further validate the importance of NCS1 for cell survival and motility and provide evidence that NCS1 modulates signaling pathways important for cancer progression. Through the use of NCS1 KO cells, we showed opposite responses when compared to previous studies with NCS1 OE (Apasu *et al.*, 2019; Moore *et al.*, 2017). Specifically, NCS1 KO led to decreased cell survival and motility compared to WT cells. Our data indicate that NCS1 affects cell survival and motility through the modulation of Ca^2+^ signaling and consequent regulation of Akt activation. We found that NCS1 regulates Ca^2+^ homeostasis and signaling by maintaining low intracellular Ca^2+^ levels under resting conditions and by enhancing InsP3‐dependent ER Ca^2+^ release. Additionally, we found that NCS1 increases Akt signaling in a Ca^2+^‐dependent manner. Both Ca^2+^ signaling and Akt are well‐established modulators of cell survival and motility (Monteith *et al.*, 2017; Vivanco and Sawyers, 2002). We found that under resting conditions, NCS1 KO cells have higher intracellular Ca^2+^ levels than WT cells, and that this increase can be rescued by the intracellular Ca^2+^ chelator BAPTA‐AM, indicating that NCS1 is crucial for maintaining low resting Ca^2+^ concentrations, which may be explained through two mechanisms. First, it is possible that NCS1 acts as a Ca^2+^ buffer. Typically, Ca^2+^ buffers have low Ca^2+^‐binding affinity_,_ are highly abundant in the cytosol, chelate Ca^2+^, and often terminate Ca^2+^ signals upon binding of Ca^2+^ (Schwaller, 2010). Second, it is also possible that NCS1 acts as a Ca^2+^‐sensor protein. Ca^2+^‐sensor proteins have a high affinity to Ca^2+^ and, upon binding of Ca^2+^, change their confirmation and interact with effector proteins (Burgoyne, 2007). Because NCS1 is a high‐affinity, low‐capacity Ca^2+^‐binding protein (Burgoyne, 2007), it is likely that NCS1 maintains low resting Ca^2+^ conditions through its interactions with other associated effector proteins, rather than acting as a Ca^2+^ buffer. Additionally, we demonstrated that under resting conditions, the survival‐ and migration‐promoting Akt pathway (Song *et al.*, 2005; Wang *et al.*, 2012; Xue and Hemmings, 2013) is less active in NCS1 KO compared to WT cells and shows enhanced activity in NCS1 OE cells. Buffering intracellular Ca^2+^ with BAPTA‐AM increases Akt activity in NCS1 KO cells but not in WT or NCS1 OE cells. These results indicate that the increased resting Ca^2+^ levels in NCS1 KO cells lead to decreased Akt activity. We cannot completely rule out the possibility that BAPTA‐AM affects Akt activity through another mechanism than buffering Ca^2+^. However, as BAPTA‐AM is widely used as a Ca^2+^ chelator and we were able to validate its Ca^2+^‐buffering function in our cells, it is most likely that Akt activity is increased due to Ca^2+^ buffering. Consistent with our findings, a previous study showed that prolonged high intracellular Ca^2+^ increases the expression of the endogenous Akt inhibitor PP2Ac, leading to less Akt activity. Conversely, Ca^2+^ buffering with BAPTA‐AM decreases PP2Ac expression and increases Akt activity (Yasuoka *et al.*, 2004). Analysis of the RNA expression data from human noncancerous and breast cancer tissue revealed a negative correlation between NCS1 expression and PP2Ac expression (Fig. S4A), supporting the hypothesis that low NCS1 levels lead to higher Ca^2+^ levels, consequently increasing PP2Ac expression and less Akt activity. However, we could not confirm this correlation through immunoblotting for PP2Ac (Fig. S4B). Most likely, western blot was not sensitive enough to detect a difference between the two genotypes. Because of this, we cannot conclusively claim that enhanced PP2Ac expression is the reason for decreased Akt activity in NCS1 KO cells. It does remain an intriguing possibility, however, and will require further studies to elucidate this mechanism more thoroughly. It should be noted that the literature surrounding the effect of intracellular Ca^2+^ on Akt activity remains controversial. There are studies supporting our results, showing that high intracellular Ca^2+^ levels decrease Akt phosphorylation and that buffering intracellular Ca^2+^ can increase Akt phosphorylation (Conus *et al.*, 1998; Kang *et al.*, 2017). Conversely, other studies claim that increased intracellular Ca^2+^ promotes Akt activity (Divolis *et al.*, 2016; Wang *et al.*, 2017). Certainly, there are underlying mechanisms in the regulation of Akt that remain incompletely understood and that require further studies in the future. In our hands, we conclude that increased intracellular resting Ca^2+^ levels can explain the observed decrease in Akt activity in NCS1 KO cells, which also explains these cells' decreased cell survival and motility.

Besides the effect of resting Ca^2+^ on cancer‐related pathways, dysregulated Ca^2+^ release from internal stores such as the ER can influence cancer development and progression (Monteith *et al.*, 2017). We observed a diminished Ca^2+^ response to the InsP3‐generating external stimulus UTP in MDA‐MB231 NCS1 KO cells, indicating reduced Ca^2+^ release from the ER in the absence of NCS1. Another study reported that NCS1 silencing in MDA‐MB231 cells has no major effect on ER Ca^2+^ signaling (Bong *et al.*, 2019). However, the difference between our results can be attributed to differences in the experimental setup. Specifically, the previous study used a model of transient NCS1 silencing, which incompletely deleted NCS1 expression which would markedly reduce the ability to detect NCS1‐dependent effects on Ca^2+^ signaling. Our study used cell lines with stable KO and OE of NCS1. In addition, the previous study used high concentrations of the protease trypsin and ATP to induce InsP3‐mediated ER Ca^2+^ release, whereas this study used UTP, an agonist optimized for this cell line, to tease out differences among genotypes. Furthermore, Ca^2+^ transients in the previous study were recorded using a plate reader, which has well‐documented limitations and is less sensitive compared to single cell‐based Ca^2+^ imaging (Heusinkveld and Westerink, 2011). By using an optimized experimental setup to study the effects of long‐term NCS1 deletion on Ca^2+^ signaling, using UTP as an agonist to induce InsP3‐mediated Ca^2+^ release, and utilizing single cell‐based Ca^2+^ imaging, our data identify NCS1 as an important regulator of the InsP3‐mediated Ca^2+^ response.

We suggest that the findings in our study have implications on tumor aggressiveness. Previously, it was shown that NCS1 preferentially localizes to the leading edge of migrating cells (Apasu *et al.*, 2019), which have a rear‐to‐front Ca^2+^ gradient with high levels of Ca^2+^ at the rear and lower Ca^2+^ at the leading edge. Disrupted Ca^2+^ homeostasis can therefore lead to abnormal migration of cancer cells (Tsai *et al.*, 2015; Wei *et al.*, 2009). Because NCS1 keeps Ca^2+^ concentrations low under resting conditions and upon stimulus enhances ER Ca^2+^ release, we suggest that NCS1 facilitates cell movement by regulating Ca^2+^ signals at the leading edge.

## 5 Conclusion

Overall, our study is the first to elucidate an underlying signaling mechanism through which the Ca^2+^‐binding protein NCS1 influences tumor aggressiveness and progression (Fig. 8). We identified NCS1 as a stress response protein that is up‐regulated by exogenous stressors that activate the transcription factor NFκB, a central regulator of the cell stress response. We found that this mechanism of NCS1 up‐regulation is conserved in the pathophysiology of human breast cancer, and we showed that NCS1 has essential functions for cell survival and migration through altering intracellular Ca^2+^ signaling and Akt signaling. The novel finding that extracellular stressors lead to NCS1 up‐regulation helps us understand how high NCS1 expression leads to more aggressive behavior of tumor cells and worse patient outcome (Apasu *et al.*, 2019; Moore *et al.*, 2017; Schuette *et al.*, 2018). In conclusion, we describe a novel role for NCS1 as stress response protein linking the tumor microenvironment and cancer progression. As NCS1 is ubiquitously expressed, our findings raise the possibility that NCS1 also contributes to other disease states that are related to the activation of the cellular stress response through NFκB and Ca^2+^ signaling.

## Conflict of interest

HKG, TTF, and JAS received a scholarship from the German Academic Scholarship Foundation. NIH support is acknowledged: GM007324, 5P01DK057751 (BEE), and F31DK118836 (ALB) supported this work. We acknowledge the use of the Yale Center for Cellular and Molecular Imaging (NIH grants 5P30DK034989 and OD020142). BEE is a founder of Osmol Therapeutics, a company that is targeting NCS1 for therapeutic purposes. The other authors declare no conflicts of interest.

## Author contributions

HKG and BEE designed the study. HKG performed the majority of the experiments, analyzed the results, and drafted the manuscript. TTF, ALB, and JAS performed additional experiments. HKG, TTF, ALB, and BEE edited the manuscript. All authors edited and agreed to the final version of the manuscript.

## Supporting information


**Fig S1.** NCS1 mRNA expression does not change with all cell stressors.

**Fig S2.** Wound healing assay.

**Fig S3.** MDA‐MB231 cells stably overexpressing NCS1.

**Fig S4.** Effect of NCS1 expression on endogenous Akt inhibitor PP2Ac.

Fig S1‐S4

## Data Availability

All specialized reagents generated for this study are available by contacting the corresponding author.
