# Supplementary figures and images for: Neuronal Calcium Sensor 1 is up‐regulated in response to stress to promote cell survival and motility in cancer cells
Source: Mol Oncol. 2020 Apr 28;14(6):1134–51. doi: 10.1002/1878-0261.12678 (PMC7266285; doi:10.1002/1878-0261.12678)

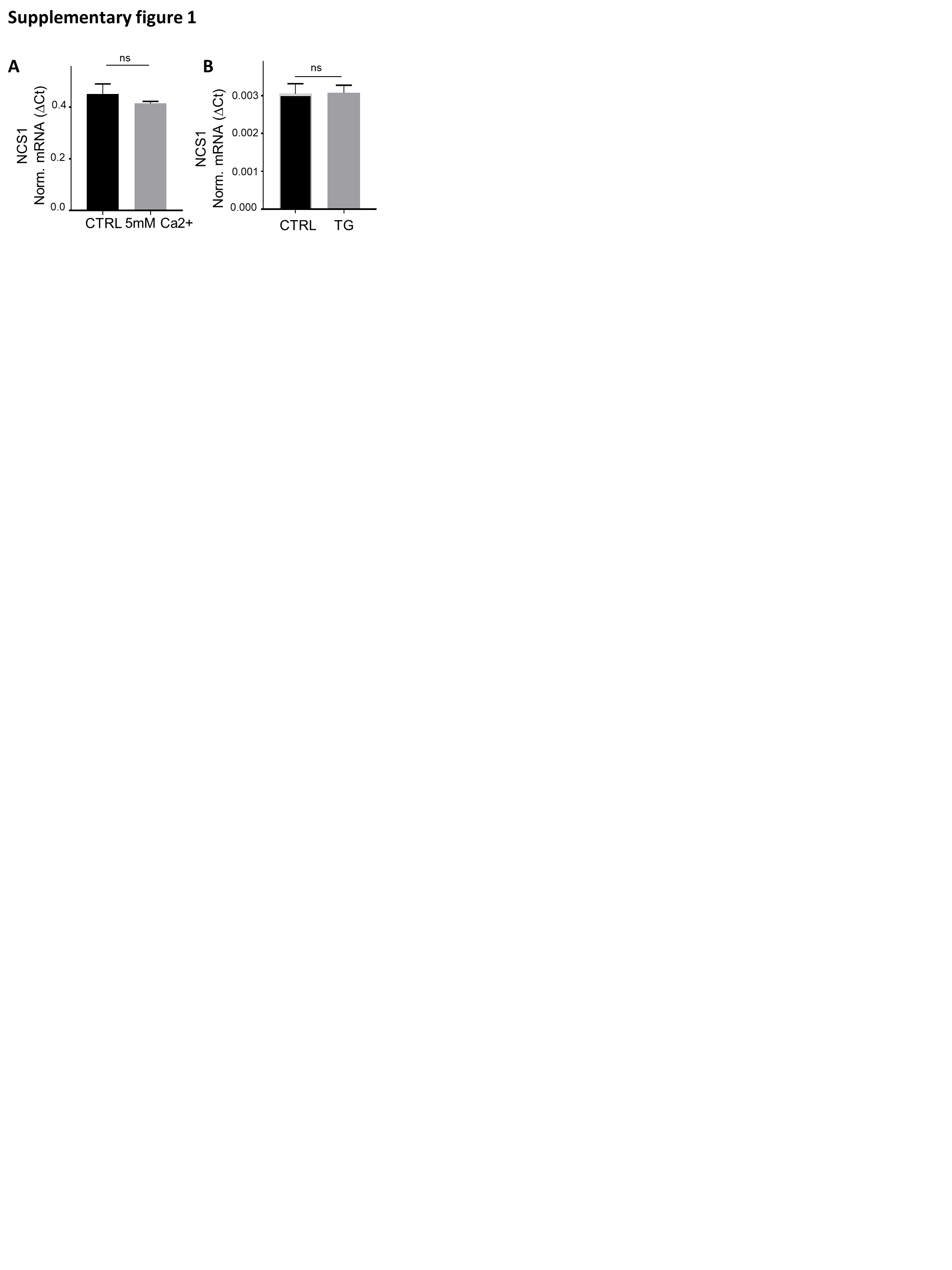

Supplement: Supplementary file 1 — Fig S1. NCS1 mRNA expression does not change with all cell stressors. [file MOL2-14-1134-s001.tif]

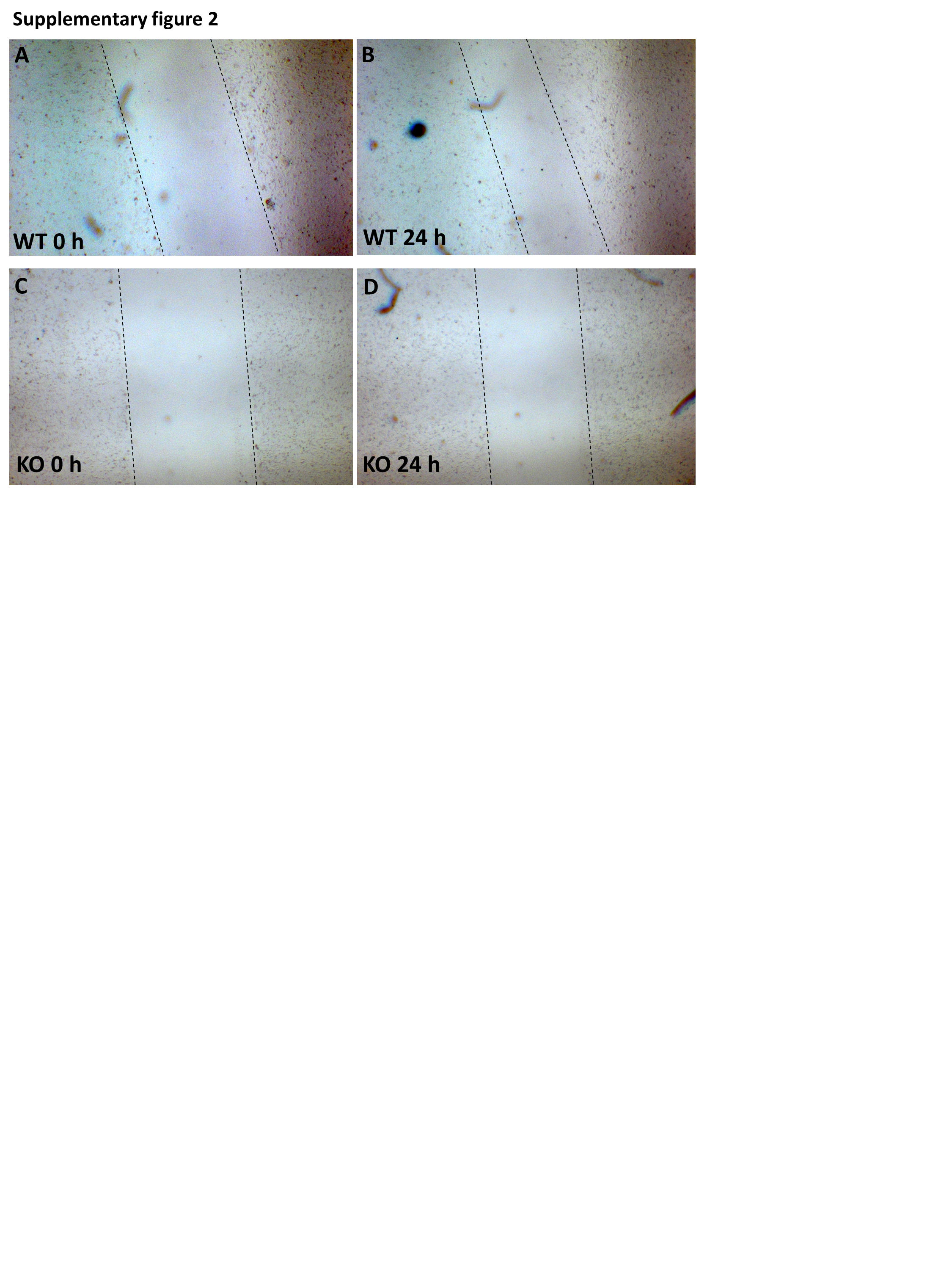

Supplement: Supplementary file 2 — Fig S2. Wound healing assay. [file MOL2-14-1134-s002.tif]

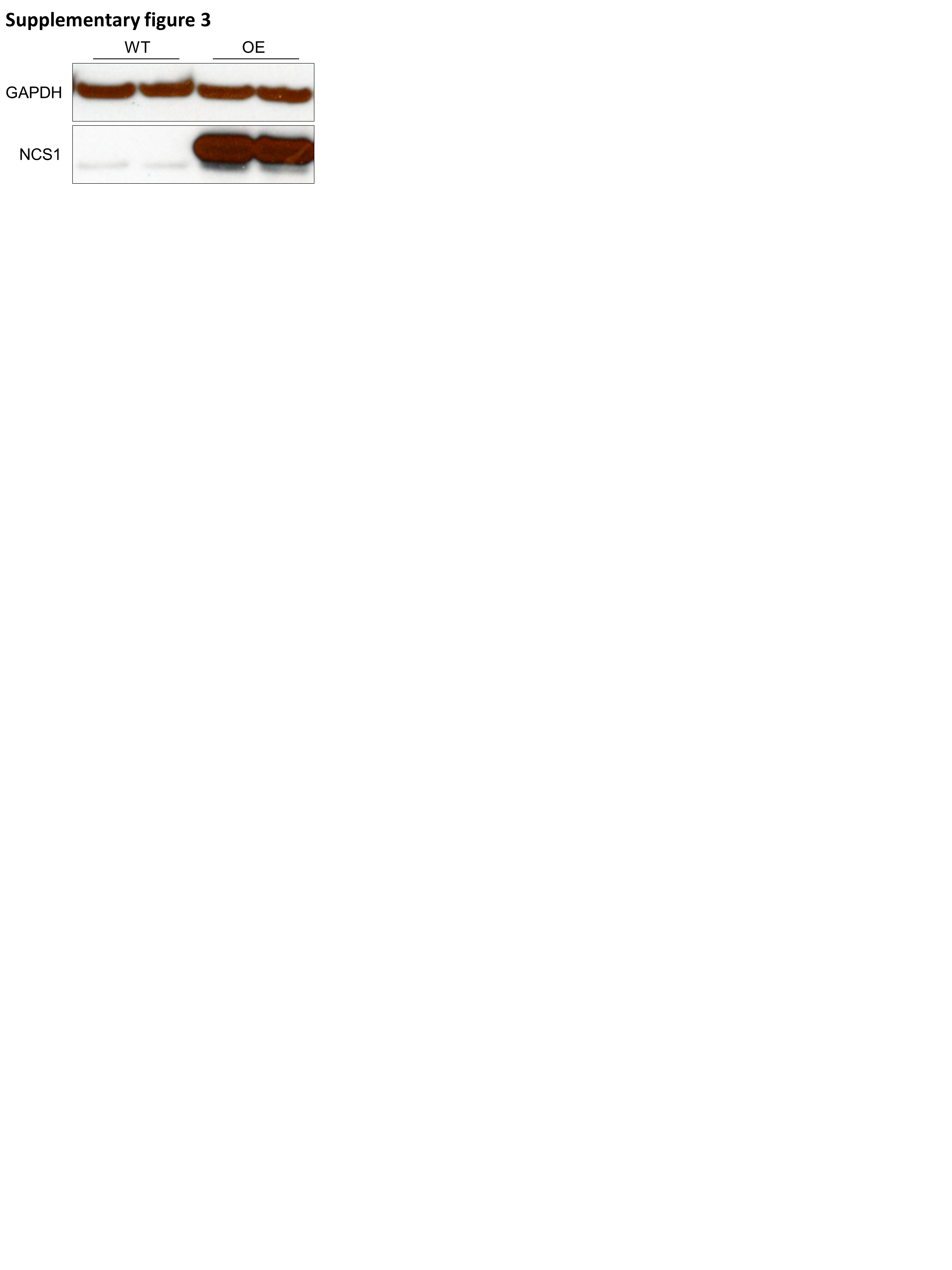

Supplement: Supplementary file 3 — Fig S3. MDA‐MB231 cells stably overexpressing NCS1. [file MOL2-14-1134-s003.tif]

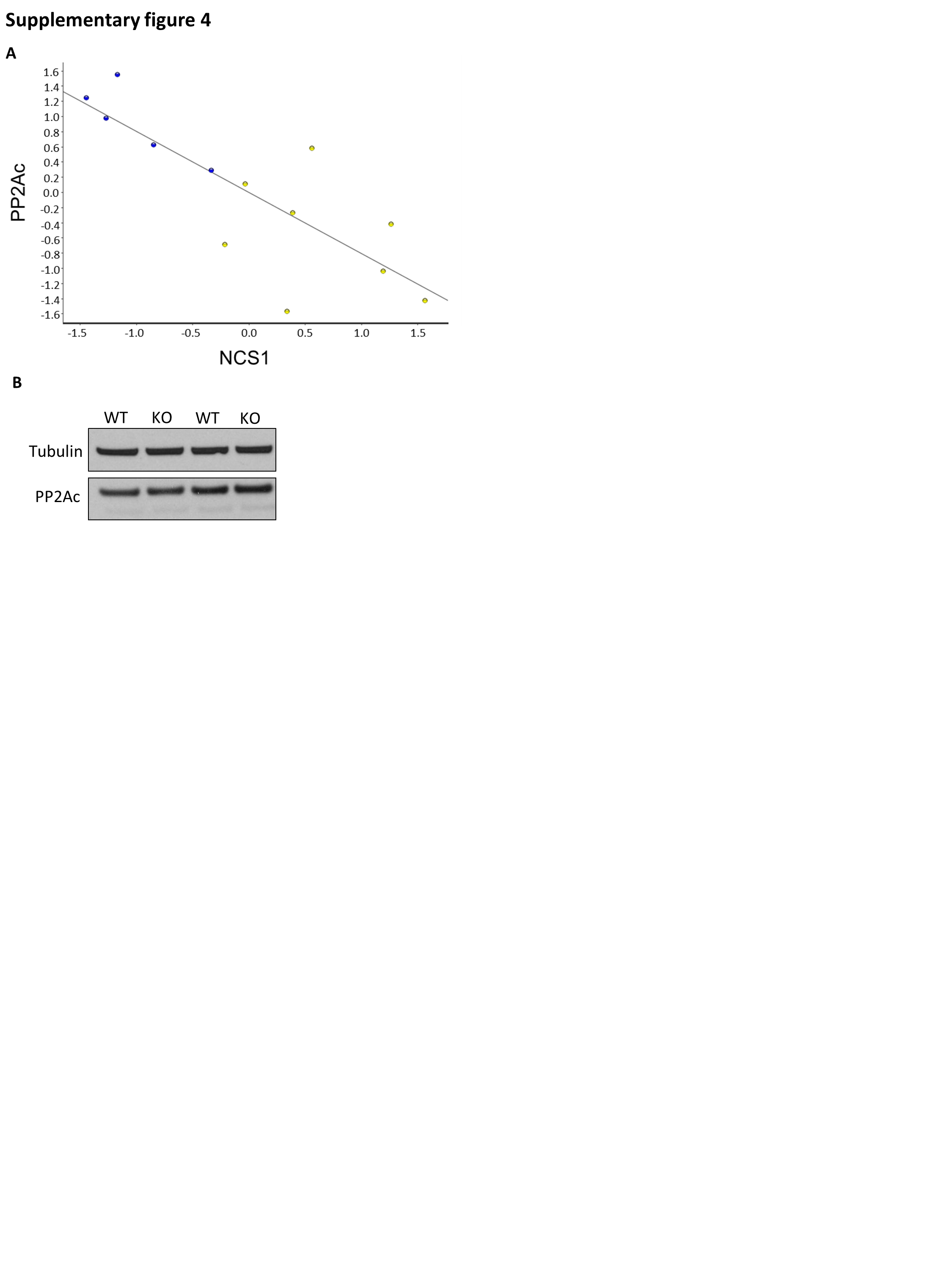

Supplement: Supplementary file 4 — Fig S4. Effect of NCS1 expression on endogenous Akt inhibitor PP2Ac. [file MOL2-14-1134-s004.tif]
