# Supplementary material for: Neuronal Calcium Sensor 1 is up‐regulated in response to stress to promote cell survival and motility in cancer cells
Source: Mol Oncol. 2020 Apr 28;14(6):1134–51. doi: 10.1002/1878-0261.12678 (PMC7266285; doi:10.1002/1878-0261.12678)
Supplement: Supplementary file 5 — Fig S1‐S4 [file MOL2-14-1134-s005.docx]

**SUPPORTING INFORMATION**

**
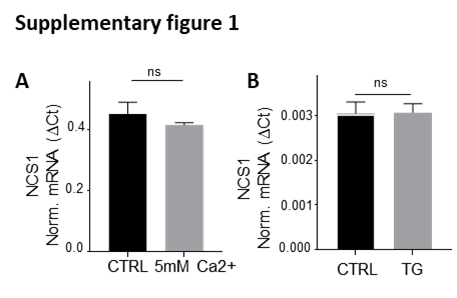
**

**Supplementary figure 1: NCS1 mRNA expression does not change with all cell stressors. A**, **B**) High extracellular Ca^2+^ and Thapsigargin (TG) did not change *NCS1* mRNA expression compared to DMSO (CTRL) treatments in SHSY5Y cells. **A**) Quantitative real-time PCR of SHSY5Y cells treated with additional 5 mM extracellular Ca^2+^ for 24 hours. **B**) Quantitative real-time PCR of SHSY5Y cells treated with 1 μM TG for 24 hours. *NCS1* mRNA levels are normalized to 18S rRNA.


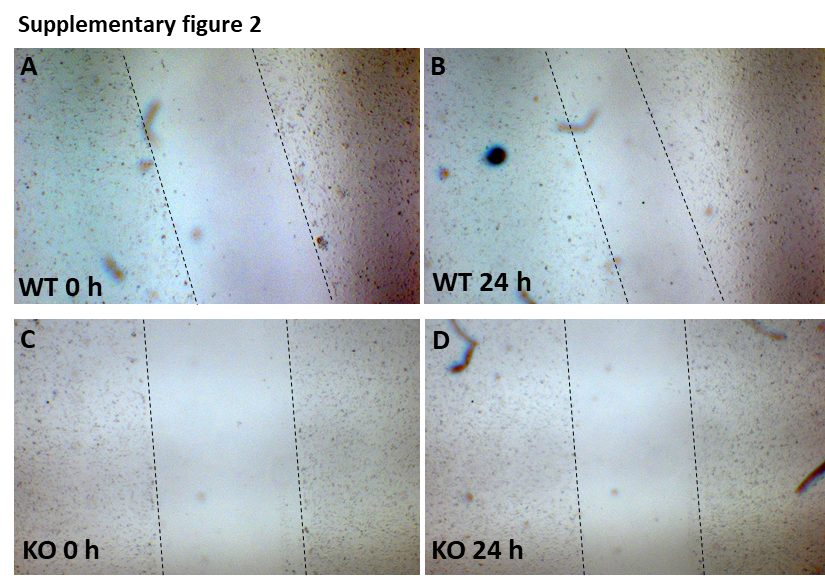


**Supplementary figure 2: Wound healing assay. A, B**) Example images of WT cells (A) directly after and (B) 24 hours after wound induction. **C** and **D**) Example images of NCS1 KO cells (C) directly after and (D) 24 hours after wound induction.


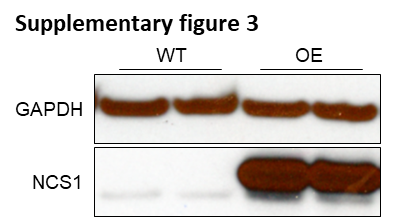


**Supplementary figure 3: MDA-MB231 cells stably overexpressing NCS1.** Western blot analysis of WT and NCS1 OE MDA-MB231 cells showing NCS1 overexpression. NCS1 OE cells show two bands for NCS1, the lower band is endogenous NCS1 and the upper band the stably transfected NCS1. The increased molecular weight is explained by a human influenza hemagglutinin (HA)-tag at the c-terminus of NCS1.


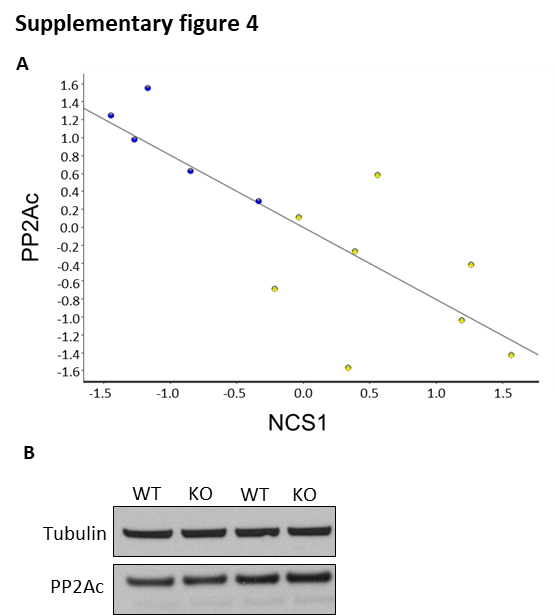


**Supplementary figure 4: Effect of NCS1 expression on endogenous Akt inhibitor PP2Ac. A**) RNA microarray analysis of human breast cancer tissue and normal breast tissue reveals a negative correlation between the endogenous Akt inhibitor PP2Ac and NCS1. Yellow dots represent breast cancer tissue with high NCS1 expression and low PP2Ac expression, blue dot represent normal breast tissue with high PP2Ac expression and low NCS1 expression. **B**) Western Blot analysis of PP2Ac expression in MDA-MB231 WT compared to NCS1 KO cells showing no difference in PP2Ac expression between the genotypes.
